# A Weak-Fluorine-Bond Molecule Stabilizes Hard Carbon Anodes for Practical Sodium-Ion Batteries

**DOI:** 10.1021/acsnano.5c10983

**Published:** 2025-08-16

**Authors:** Yaqi Liao, Han Liu, Yangqian Zhang, Jiayi Yang, Haijin Ji, Donghai Wang, Lixia Yuan, Yunhui Huang, Yang Ren

**Affiliations:** † Department of Physics, JC STEM Lab of Energy and Materials Physics, 53025 City University of Hong Kong, Hong Kong 999077, P. R. China; ‡ State Key Laboratory of Material Processing and Die and Mold Technology, School of Materials Science and Engineering, 12443 Huazhong University of Science and Technology, Wuhan 430074, China; § School of Renewable Energy, 12462 Hohai University, Nanjing 211100, China; ∥ Shenzhen Research Institute, 53025 City University of Hong Kong, Shenzhen 518057, P. R. China

**Keywords:** solid-electrolyte interphases, sodium-ion batteries, hard carbon anodes, weak-fluorine-bond molecules, preferential reductions

## Abstract

Solid-electrolyte interphase (SEI) is essential for improving the cycling stability in sodium-ion batteries (SIBs) by preventing direct contact between electrolytes and hard carbon (HC) anodes. Conventional C–F bond molecules like fluoroethylene carbonate (FEC) show poor SEI formation due to early sodium-ion adsorption on HC, delaying additive reduction. Herein, methyl 2, 2-difluoro-2-(fluorosulfonyl) acetate (MDFA), a weak-fluorine-bond molecule, is proposed to facilitate early SEI formation and suppress parasitic reactions. The strong electron-withdrawing O=S=O group destabilizes the S–F bond, enabling preferential reduction of MDFA and formation of inorganic SEI components that enhance ionic conductivity and accelerate interfacial charge transfer. As a result, the HC with MDFA shows over 5000 stable cycles and delivers a high capacity of 252 mAh g^–1^ at 5 C, outperforming 108 mAh g^–1^ of that with FEC. A 4.6 Ah pouch cell with MDFA enables 89.3% capacity retention after 1000 cycles. These findings provide valuable insights into fluorine-bond chemistry for the electrolyte additive design in long-life SIBs.

## Graphical abstract

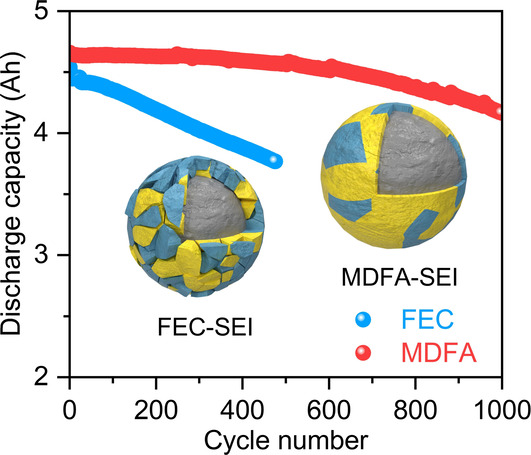

## Introduction

Sodium-ion batteries (SIBs) emerge as a promising alternative for grid-scale energy storage owing to the abundance and low cost of sodium resources.^1^ However, traditional graphite commonly applied in lithium-ion batteries (LIBs) fails to function effectively as an anode material for high-capacity and reversible sodium-ion (Na^+^) storage.^2,3^ Although the fundamental cause behind this limitation remains unclear, graphite is increasingly being phased out as a viable anode in SIBs. The sluggish progress in developing anode materials has constrained the commercial advancement of SIBs.^4,5^ Therefore, identifying and optimizing alternative anode materials are essential to unlocking the full potential of high-performance SIBs. Hard carbon (HC) contains graphitic crystallites, closed pores, and surface defects, making it a strong candidate for sodium-ion battery anodes.^6–9^ It delivers a reversible capacity of as high as 300 mAh g^–1^.^10,11^ Additionally, many HC materials are derived from decomposing biomass materials, thereby reducing their production costs.^12,13^ However, HC remains limited by insufficient cycling durability and rate performance.^14,15^ In LIBs, graphite anodes enable the formation of a stable solid-electrolyte interphase (SEI) at low voltages, which effectively suppresses parasitic reactions within the cell.^16,17^ Although a similar SEI forms on the surface of HC anodes in SIBs, it tends to be mechanically fragile and prone to cracking during repeated cycling.^18^ Once the SEI ruptures, the exposed electrode surface undergoes continuous reactions with the electrolyte, leading to the consumption of active materials and rapid capacity decay.^19^ Thus, improving the structural integrity of the SEI is crucial to the cycling performance of SIBs.^20^ A common strategy to improve SEI properties involves increasing the fluorine content in the electrolyte formulation.^21–24^ Electrolyte additives are typically divided into two types: fluorine-containing additives and fluorine-free additives. Fluorine-containing additives can offer several key advantages over fluorine-free additives in battery electrolytes, particularly for SEI stabilization in sodium-ion batteries. Fluorine-containing additives can be decomposed to form inorganic compounds in the SEI, which enhances ionic diffusion kinetics and reduces electrolyte decomposition. However, the fluorine-free additives tend to form an organic-dominated SEI (polycarbonates, oligomers), which is less stable during long cycling. Furthermore, fluorine-rich SEIs are more stable at higher voltages, enabling compatibility with high-voltage cathodes. Fluorine-poor SEI easily degrades at lower potentials, leading to thickening impedance and huge capacity fading. For example, fluoroethylene carbonate (FEC) widely used in LIBs undergoes C–F bond cleavage during cycling, leading to the formation of lithium fluoride (LiF)-enriched SEI layers.^25,26^ The presence of these inorganic components significantly reinforces the mechanical and chemical stabilities of the SEI, improving the overall cycling durability. However, the C–F bond is among the stable fluorine bonds and remains difficult to cleave.^27^ This exceptional stability arises from several key factors: (1) The large electronegativity difference between fluorine (F) and carbon (C). Fluorine exhibits the highest electronegativity (3.98) among all elements, which results in a highly polarized and partial ionic bond with carbon. This polarization reinforces the bond strength of C–F. (2) The strong Coulombic attraction between the polarized atoms, which acts as an additional force maintaining the integrity of the bond. The electronegativity gap induces electron delocalization, creating a strong electrostatic attraction between the partially positive C atoms and the partially negative F atoms. This characteristic significantly enhances the strength and stability of the C–F bond. To achieve a more robust SEI, it is necessary to investigate alternative weak-fluorine-bond molecules. Compared with the C–F bond in FEC, the SO_2_–F group is more prone to cleavage due to the strong electron-withdrawing effect of the sulfonyl group (O=S=O), which weakens the S–F bond.^28,29^ A typical example is lithium bis­(fluorosulfonyl)­imide (LiFSI), which demonstrates better SEI-forming properties than lithium bis­(trifluoromethanesulfonyl)­imide (LiTFSI).^30–32^ The superior film-forming ability of LiFSI is attributed to the SO_2_–F bond, which decomposes more readily than the C–F bond in LiTFSI.^33^ Inspired by this observation, employing a molecule containing the SO_2_–F group as a replacement for FEC may offer a promising strategy to enhance the structural stability of the SEI on the HC surface.

The common LIBs adopt graphite as the anode material. Li^+^ generally is intercalated into the graphite layers at around 0.3 V.^34–36^ Before Li^+^ intercalation, the graphite electrode undergoes the Li^+^ desolvation process and electrolyte decomposition to format the SEI on the graphite surface at around 1.0 V. However, the reaction mechanism of the anode in SIB systems is different from the common LIB systems. HC is commonly used as the anode material in SIBs. It is well accepted that the Na^+^ storage process contains “adsorption-intercalation-filling” three steps.^11^ Na^+^ generally are adsorbed on the hard carbon surface when the cell discharges to about 1.2 V.^23,37,38^ However, the SEI is not formatted adequately in time, which leads to severe parasitic reactions between the anode and the electrolytes. Hence, the cycling performance of HC is not unsatisfactory. It is necessary to develop an electrolyte additive to manipulate the SEI early formation, reducing side reactions between the anode and electrolytes, finally improving the cycling stability of the HC anode.^39^

In this work, we propose a weak-fluorine-bond electrolyte additive, methyl 2,2-difluoro-2-(fluorosulfonyl)­acetate (MDFA), to replace the traditional FEC additive and actively modulate the chemical components of the SEI layer on the HC anode surface. In contrast to the C–F bond in FEC, the SO_2_–F bond in MDFA endows it with higher reducibility. As a result, MDFA undergoes preferential reduction on the anode surface, contributing to the formation of inorganic-rich SEI and effectively suppressing excessive SEI growth and parasitic reactions within the cells. Accordingly, the HC electrode with MDFA achieves a high specific capacity of 252 mAh g^–1^ at 5 C along with good cycling stability over 5000 cycles. In contrast, the HC electrode with FEC exhibits a much lower capacity of only 115 mAh g^–1^ at 5 C and suffers from rapid capacity degradation. Sodium-ion pouch cells were assembled to assess the influence of FEC and MDFA additives. The pouch cell with MDFA delivered high initial and reversible discharge capacities of 4.65 Ah and 4.16 Ah after 1000 cycles, achieving a capacity retention of 89.3%. In contrast, the FEC-based cell retained only 82.5% of its initial capacity after 470 cycles. Ultrasonic scanning and infrared imaging revealed fewer side reactions in pouch cells with MDFA. This research presents a feasible pathway for additive design aimed at controlling SEI composition and stability, thereby extending the cycle life of SIBs.

## Results and Discussion

Figure 1 schematically illustrates the SEI formation and evolution processes on the HC surface using FEC and MDFA as electrolyte additives. In the Na/HC cell, Na^+^ adsorption on the HC surface begins at approximately 1.2 V. As the potential decreases to around 1.0 V during discharge, solvent and anion reductions occur, initiating SEI formation on the HC surface. Because Na^+^ adsorption occurs prior to electrolyte reduction, numerous parasitic reactions can occur at the interface. Therefore, it is essential to induce SEI formation on the HC anode before the Na^+^ adsorption begins. The reduction potential of an electrolyte is determined by its components, including salts, solvents, and additives. Among these, incorporating a film-forming additive is more commercially viable than replacing solvents or salts due to its lower cost and ease of implementation. The film-forming functional groups in FEC and MDFA are the C–F and SO_2_–F bonds, respectively. Due to the high stability of the C–F bond, FEC typically decomposes only after Na^+^ adsorption, resulting in a nonuniform SEI that continuously grows during cycling (Figure 1a). Compared with the strong C–F bond, the SO_2_–F bond is more susceptible to cleavage due to the electron-withdrawing effect of the O=S=O group, which destabilizes the S–F bond. As a result, MDFA exhibits a reduction potential higher than the Na^+^ adsorption potential. Before Na^+^ intercalates into the HC, MDFA undergoes reduction to form sodium fluoride (NaF) as part of the SEI, which enhances both the ionic conductivity and mechanical robustness. By the formation of a stable SEI prior to Na^+^ intercalation, parasitic reactions at the HC/electrolyte interface are minimized, contributing to improved long-term cycling performance (Figure 1b).

**Figure 1:**
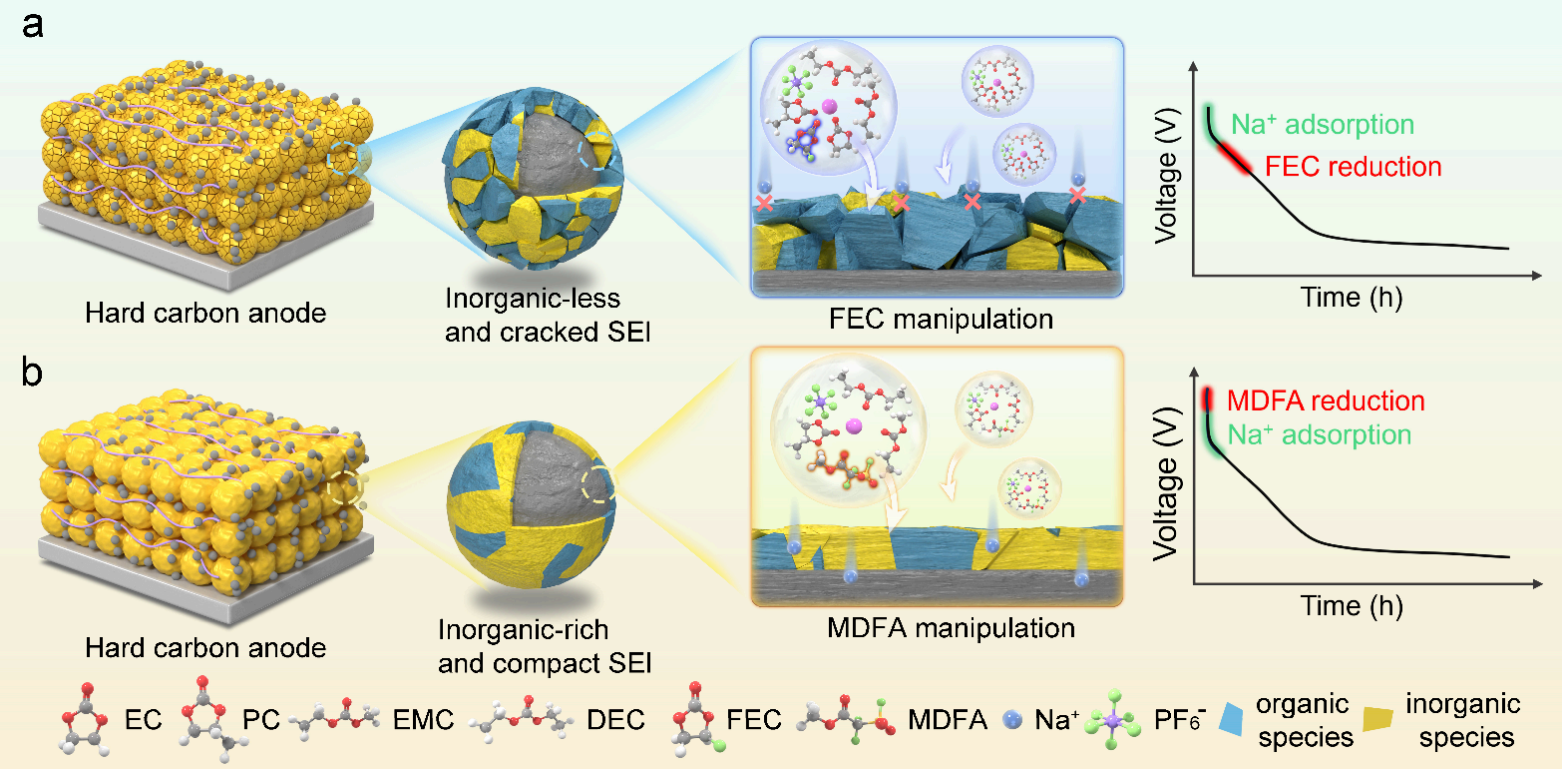
Schematic illustrations of the SEI formation and growth process on the HC surface with (a) FEC and (b) MDFA additives.

The molecular structures of FEC and MDFA are exhibited in Figure S1. Figure 2a illustrates the highest occupied orbital and the lowest unoccupied orbital energy levels of the solvents and additives in the electrolyte, serving as indicators of their respective oxidation and reduction potentials. The LUMO levels of ethylene carbonate (EC), propylene carbonate (PC), ethyl methyl carbonate (EMC), diethyl carbonate (DEC), FEC, and MDFA free molecules are −6.26, −6.16, −5.95, −5.88, −6.73, and −7.13 eV, respectively. The LUMO level of MDFA is lower than those of other molecules, indicating its higher tendency to undergo reduction on the HC anode and contribute to the formation of a stable SEI. After coordination with Na^+^, the LUMO levels of Na^+^-EC, Na^+^-PC, Na^+^-EMC, Na^+^-DEC, and Na^+^-FEC decrease to −5.18, −5.11, −5.51, −5.45, and −5.31 eV, respectively. MDFA presents four coordination sites with Na^+^, corresponding to LUMO levels of −7.43, −7.37, −6.17, and −7.37 eV for Na^+^-MDFA1, Na^+^-MDFA2, Na^+^-MDFA3, and Na^+^-MDFA4, respectively. Even when coordinated with Na^+^, MDFA remains more susceptible to reduction. As shown in Figure 2b, the electrostatic potential maps reveal pronounced electron cloud redistribution around the C–F and SO_2_–F bonds, attributed to the strong electron-withdrawing ability of fluorine. The bond length of the C–F bond of the FEC is around 1.37 Å. The C–F and S–F bonds of MDFA are calculated as 1.33 and 1.59 Å, respectively (Figure S2). The longer bond length of the S–F bond of MDFA implies easier bond dissociation. Electron location function (ELF) also confirms the electron transference of FEC and MDFA (Figure S3). The calculated bond dissociation energies reveal that the SO_2_–F bond possesses a lower dissociation energy of 404 kJ mol^–1^ compared to 480 kJ mol^–1^ for the C–F bond in FEC, 468 kJ mol^–1^ for the C–F1 bond in MDFA, and 460 kJ mol^–1^ for the C–F2 bond in MDFA (Figure 2c). Bond dissociation energy calculations confirm the ease of SO_2_–F bond cleavage in MDFA, implying that its reduction may contribute to the generation of inorganic components in the SEI. Crystal orbital overlap population (COOP) calculations were performed to quantitatively assess the bond strength (Figure 2d–g). The COOP values for the C–F bonds in FEC, C–F1, C–F2, as well as the SO_2_–F bonds in MDFA are 0.22, 0.25, 0.27, and 0.20 Ha, respectively. The lower COOP value of the SO_2_–F bond confirms its weaker bond strength, consistent with the dissociation energy results. Subsequently, the binding energies between Na^+^ and various molecules were calculated to evaluate the coordination environment of the sodium ions. The binding energies of Na^+^-EC, Na^+^-PC, Na^+^-EMC, Na^+^-DEC, Na^+^-FEC, Na^+^-MDFA1, Na^+^-MDFA2, Na^+^-MDFA3, and Na^+^-MDFA4 are −1.41, −1.44, −1.18, −1.18, −1.20, −0.55, −1.02, −0.55, and −1.02 eV, respectively (Figure S4). These results suggest that MDFA exhibits the weakest solvation ability among the various molecules. Molecular dynamics (MD) simulations were conducted to investigate the Na^+^ solvation structures in bulk electrolytes (Figure S5). Radial distribution function (RDF) and coordination number (*n*) analyses indicate that the solvation structures of Na^+^ remain largely unchanged in both FEC- and MDFA-based electrolytes. This suggests that MDFA does not significantly alter the Na^+^ coordination environment, as it primarily resides in the outermost layer of the solvation sheath. The MDFA-based electrolyte exhibits higher ionic conductivity and a broader electrochemical stability window compared to the FEC-based electrolyte (Figures S6 and S7).

**Figure 2:**
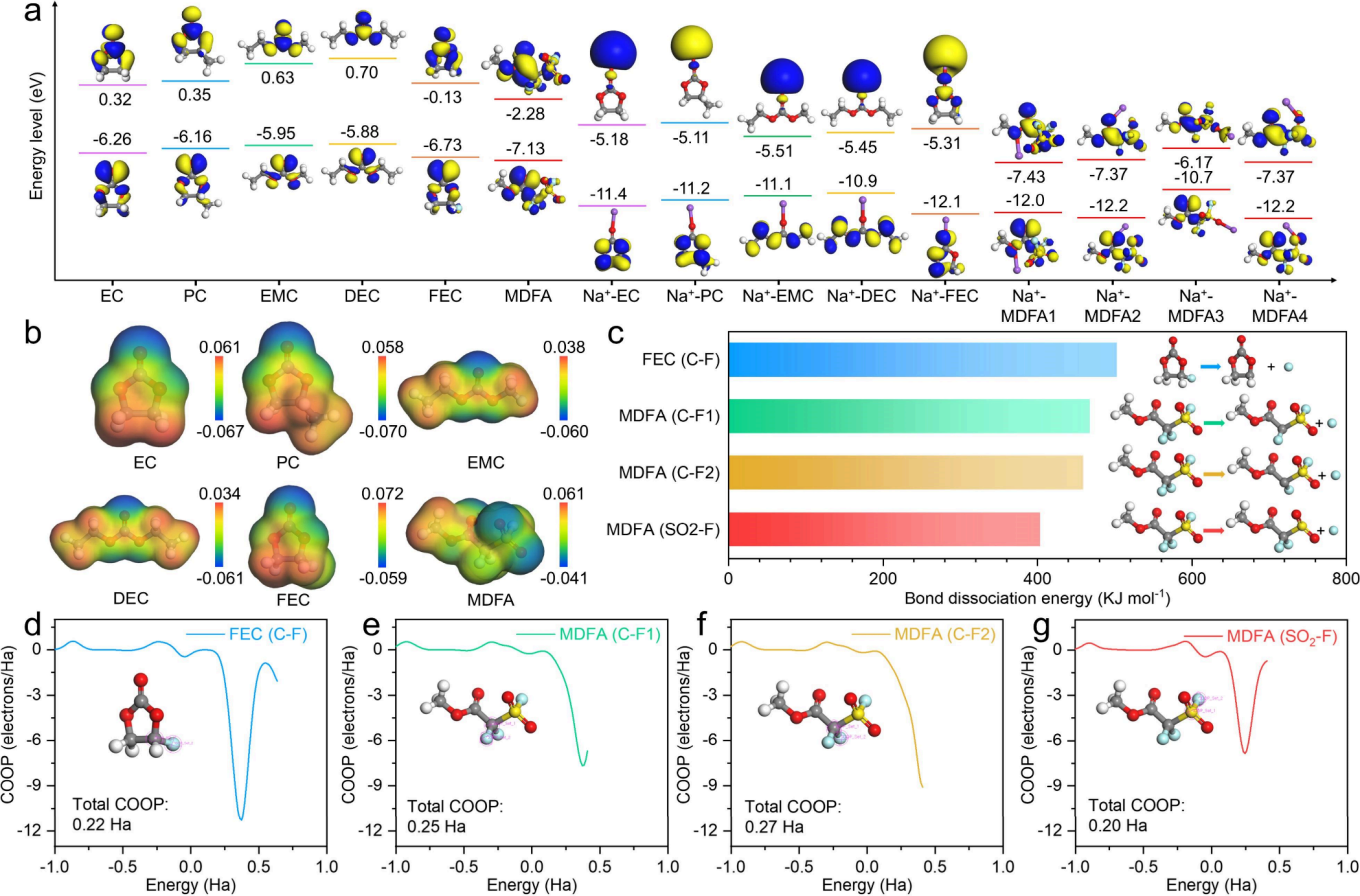
Theoretical calculations of different molecules: (a) Energy levels, (b) electrostatic potentials, and (c) chemical bond dissociation energies. Crystal orbital overlap population: (d) C–F of FEC; (e) C–F1, (f) C–F2, and (g) SO_2_–F of MDFA.

Next, half cells are assembled to evaluate the effect of FEC and MDFA on the electrochemical performance. Figure 3a,b demonstrates the cyclic voltammograms (CVs) of the FEC and MDFA cells recorded at different scan speeds. Sharp reduction/oxidation peaks at ∼0.01 and 0.1 V correspond to the Na^+^ insertion and extraction process. The MDFA-based cell exhibits higher reduction and oxidation current densities than the FEC-based cell, indicating improved reaction kinetics. Moreover, the overlapping CV curves during the initial three cycles in the MDFA-based cell reflect good electrochemical reversibility (Figure S8). The galvanostatic intermittent titration technique (GITT) further confirms enhanced Na^+^ diffusion kinetics in the MDFA-based cell (Figure S9). Based on the initial charge/discharge curves, the initial Coulombic efficiencies (ICEs) of the cells with FEC and MDFA electrolytes are 83.2 and 91.6%, respectively (Figure 3c). The increased ICE reflects the improved interfacial stability between the HC anode and electrolytes. In contrast, cells containing other MDFA-based additives demonstrate significantly lower ICEs (Figure S10). A lower concentration of MDFA may lead to nonuniform SEI formation, whereas excessive MDFA can result in an overly thick SEI layer, both of which are detrimental to efficient Na^+^ transport. The rate performances of the FEC and MDFA cells are compared in Figure 3d–f. The MDFA cell shows higher discharge capacities of 325, 317, 307, 294, 284, 272, 260, and 252 mAh g^–1^ at 0.1, 0.2, 0.5, 1, 2, 3, 4, and 5 C, respectively. In contrast, the FEC cell can deliver discharge capacities of only 312, 267, 259, 235, 177, 141, 123, and 108 mAh g^–1^ at 0.1, 0.2, 0.5, 1, 2, 3, 4, and 5 C, respectively. In addition, the MDFA-based cell exhibits a higher plateau capacity ratio, further confirming faster Na^+^ storage kinetics (Figure S11). The discharge capacity of the HC with the help of MDFA is 247 mAh g^–1^ at–20 °C, higher than the 172 mAh g^–1^ of the FEC cell (Figure S12). The MDFA-based cell also shows lower interfacial impedance compared with the FEC-based cell (Figure S13). The cycling performance of cells is also measured. At 0.5 C, the MDFA cell can cycle stably over 400 cycles, while the FEC cell delivers huge capacity decay after 300 cycles (Figures S14 and S15). As shown in Figure S16, the structure of methyl difluoroacetate (MFA) is similar to that of MDFA except without the SO_2_–F group. The HC electrode with MFA delivers high and reversible discharge capacities of 302 and 283 mAh g^–1^ after 400 cycles, respectively (Figure S17). Although the MFA molecule also has an optimizing effect on the HC anode, the electrochemical performance of the MFA cell is also inferior than that of the MDFA cell, confirming the crucial role of the SO_2_–F group. Benefiting from the MDFA-derived SEI, the HC can still cycle stably over 200 cycles, even at a high mass loading of 6 mg cm^–2^ (Figure S18). Furthermore, the MDFA cell can maintain a high discharge capacity of 109 mAh g^–1^ after 5000 cycles at 5 C, corresponding to a low capacity decay ratio of 0.009% per cycle (Figures 3g and S19). In comparison, the FEC-based cell shows a significantly lower discharge capacity of only 37 mAh g^–1^ after 5000 cycles. The HC with the MDFA additive has plateau capacity ratios higher than those of the FEC cell during long cycling, suggesting that MDFA can accelerate the sodium-ion storage reaction kinetics (Figure S20). The digital photo of the MDFA electrolyte after 1 week of aging is shown in Figure S21. The electrolyte remained colorless and transparent during aging. The cell with the aged electrolyte still delivers good cycling stability (Figure S22). Scanning electron microscopy (SEM) was employed to examine the microstructural evolution of the HC anode before and after cycling (Figure S23). The HC anode shows a clean surface before cycling. After cycling in the FEC-containing electrolyte, the surface becomes significantly roughened, indicating continuous SEI growth. In contrast, the HC anode cycled in the MDFA-based electrolyte retains a clean surface, suggesting more stable SEI formation. The electrochemical performance evaluations confirm that MDFA substantially enhances both the rate capability and cycling stability of HC electrodes primarily due to the formation of a stable MDFA-modulated interfacial layer.

**Figure 3:**
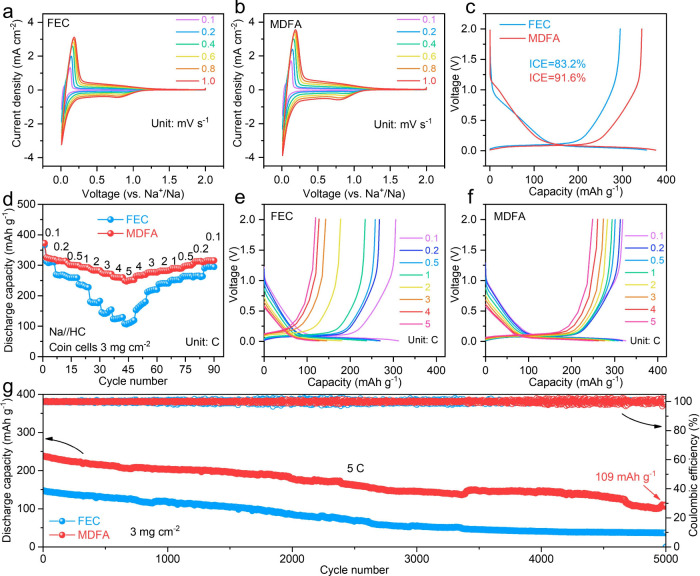
Electrochemical tests of Na//HC coin cells. The cyclic voltammograms of (a) FEC and (b) MDFA cells. (c) Initial discharging/charging curves of cells. (d) Rate performance comparison of coin cells and corresponding curves at different rates of (e) FEC and (f) MDFA cells. (g) Cycling stability comparison of cells with FEC and MDFA additives.

The electrochemical performance of HC electrodes, particularly their rate performance and long-term stability, is closely linked to the morphology and chemical composition of the SEI layer formed during cycling. Before cycling, the HC particle had a clean surface (Figure S24). As shown in Figure 4a,b, the thickness of SEI formed in the MDFA electrolyte of 8 nm is much thinner than that in the FEC electrolyte of 12 nm. The reduced interphase thickness contributes to lower ionic resistance, facilitating rapid Na^+^ transport and superior high-rate performance. To further investigate the chemical composition of the SEI, X-ray photoelectron spectroscopy (XPS) depth profiling was performed (Figure S25). The atomic ratios of the SEI components at various depths are presented in Figure 4c,d. The elevated fluorine content in the MDFA electrolyte arises from the facile decomposition of the SO_2_–F bond in MDFA. The C 1s spectrum is used to evaluate the presence of organic species commonly produced through solvent breakdown. The peaks at 285.5, 286.5, 287.9, 285.5, 290, and 291 eV belong to the C–C, C–O, C=O, O=C–O, and CO_3_^2–^ chemical bonds, respectively (Figure 4e,g). The C–F and Na–F bonds are at 685.7 and 690.3 eV, respectively (Figure 4f,h). These results indicate that the SEI is composed of both organic and inorganic species, with the inorganic components facilitating enhanced ionic diffusion kinetics. In the Na 1s and F 1s spectra, the SEI formed in the MDFA-based electrolyte exhibits a significantly higher content of NaF and Na_2_O compared to that in the FEC-based electrolyte (Figure S26). Time-of-flight secondary-ion mass spectrometry (TOF-SIMS) was further employed to analyze the spatial distribution of SEI components (Figures 4i,j, S27, and S28). The C^–^, C_2_H^–^, CH_3_O_2_^–^, and C_2_HOCO_2_^–^ fragments are associated with organic SEI components, whereas the F^–^ fragment is indicative of inorganic species such as NaF. The F^–^ signal intensity in the MDFA-derived SEI is significantly higher than that in the FEC-derived SEI, suggesting that MDFA facilitates the formation of an inorganic-rich SEI, thereby enhancing its compactness.

**Figure 4:**
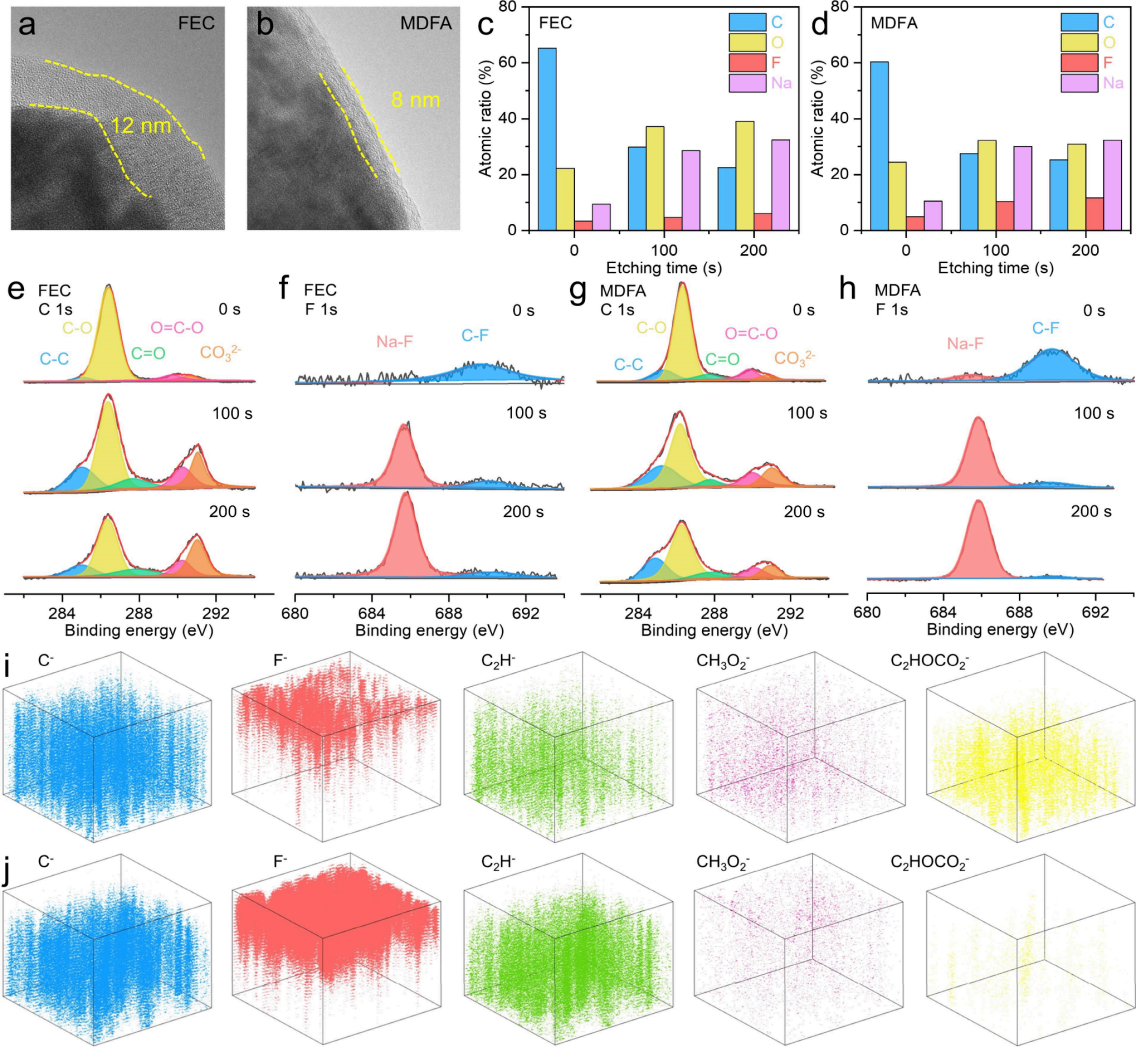
Structure and chemical composition analysis of the SEI. TEM images of cycled HC electrodes in (a) FEC and (b) MDFA electrolytes. The SEI element atomic ratios in (c) FEC and (d) MDFA electrolytes. XPS analysis of (e) C 1s and (f) F 1s spectra in the FEC electrolyte. XPS analysis of (g) C 1s and (h) F 1s spectra in the MDFA electrolyte. TOF-SIMS results of the SEI in (i) FEC and (j) MDFA electrolytes. Scale area is 100 × 100 μm.

Finally, Ah-level sodium-ion pouch cells are assembled to demonstrate the practical application potential of MDFA. Layered oxides (NNFMO) are chosen as cathode materials because they combine the advantages of higher energy density over polyanionic systems and more stable cycling performance compared to Prussian-blue-based cathodes. The structural and morphological features of both the cathode and the anode are presented in Figures S29 and S30,) respectively. Detailed assembly parameters of the pouch cells are listed in Table S1. Figure 5a,b depicts the initial formation curves of the pouch cells. During the initial formation process, a distinct voltage plateau appears at approximately 1.0 V in the MDFA-containing cell, indicating the preferential reduction of MDFA. In contrast, no such plateau is observed in the FEC-based cell, suggesting the absence of early stage reduction. Self-discharge tests were conducted to assess the long-term storage stability. After 1 month of aging, the pouch cell with MDFA maintained a higher open-circuit voltage compared to that of the FEC counterpart, demonstrating superior storage stability (Figure 5c). The rate capability of the pouch cells was evaluated over a current range from 0.5 to 10 C (Figure 5d). The pouch cell with the FEC electrolyte shows 4.48, 4.38, 4.24, 4.15, 4.00, and 3.24 Ah at 0.5, 1, 2, 3, 5, and 10 C, respectively (Figure 5e). However, the pouch cell with the MDFA electrolyte delivers 4.65, 4.56, 4.45, 4.37, 4.26, and 3.87 Ah at 0.5, 1, 2, 3, 5, and 10 C, respectively (Figure 5f). This enhancement is primarily attributed to the formation of a more ionically conductive SEI layer promoted by MDFA, which facilitates faster Na^+^ transport at high rates. Figure 5g shows the cycling performance of the pouch cells. The pouch cell with MDFA displays high initial and reversible capacities of 4.65 and 4.16 Ah after 1000 cycles, respectively, corresponding to a low decay ratio of 0.009% per cycle (Figure S31a). In contrast, the pouch cell with FEC delivers high initial but low reversible capacities of 4.53 and 3.77 Ah after 470 cycles, respectively (Figure S31b). The average voltages of the FEC and MDFA pouch cells are maintained at ∼3.09 V (Figure S32). The energy density of the MDFA pouch cell is calculated as 133 Wh kg^–1^ (Figure S33). This rapid capacity degradation is attributed to persistent SEI cracking and reformation, along with severe parasitic reactions between the electrolyte and the anode. The pouch cells cycled in different electrolytes were disassembled for post-mortem analysis. Noticeable sodium deposition was observed on the HC anode cycled with the FEC electrolyte (Figure S34a), whereas the HC surface remained largely unchanged after cycling in the MDFA-based electrolyte (Figure S34b). Additionally, the overall cell thickness increased from 10.2 to 15.5 mm in the FEC system, while the MDFA-based cell maintained a nearly constant thickness of 10.7 mm after cycling (Figure S35). To further evaluate the temperature tolerance of pouch cells, cycling tests were conducted at 50 °C. At an elevated temperature of 50 °C, side reactions are aggravated, and the cycle life of cells is shortened. The pouch cell with MDFA shows a high capacity retention of 95% after 160 cycles compared to 86% of the FEC cell (Figures S36 and S37). At a low temperature of −20 °C, the discharge capacity is increased from 2.23 to 3.63 Ah after introducing MDFA. The discharge capacity of pouch cells is measured at a lower temperature of −40 °C. At 0.1 C, the MDFA pouch cell can show a discharge capacity of 3.73 Ah, higher than 2.75 Ah of the FEC pouch cell (Figure S38). These findings demonstrate that MDFA improves thermal adaptability and electrochemical stability of pouch cells across a wide temperature range.

**Figure 5:**
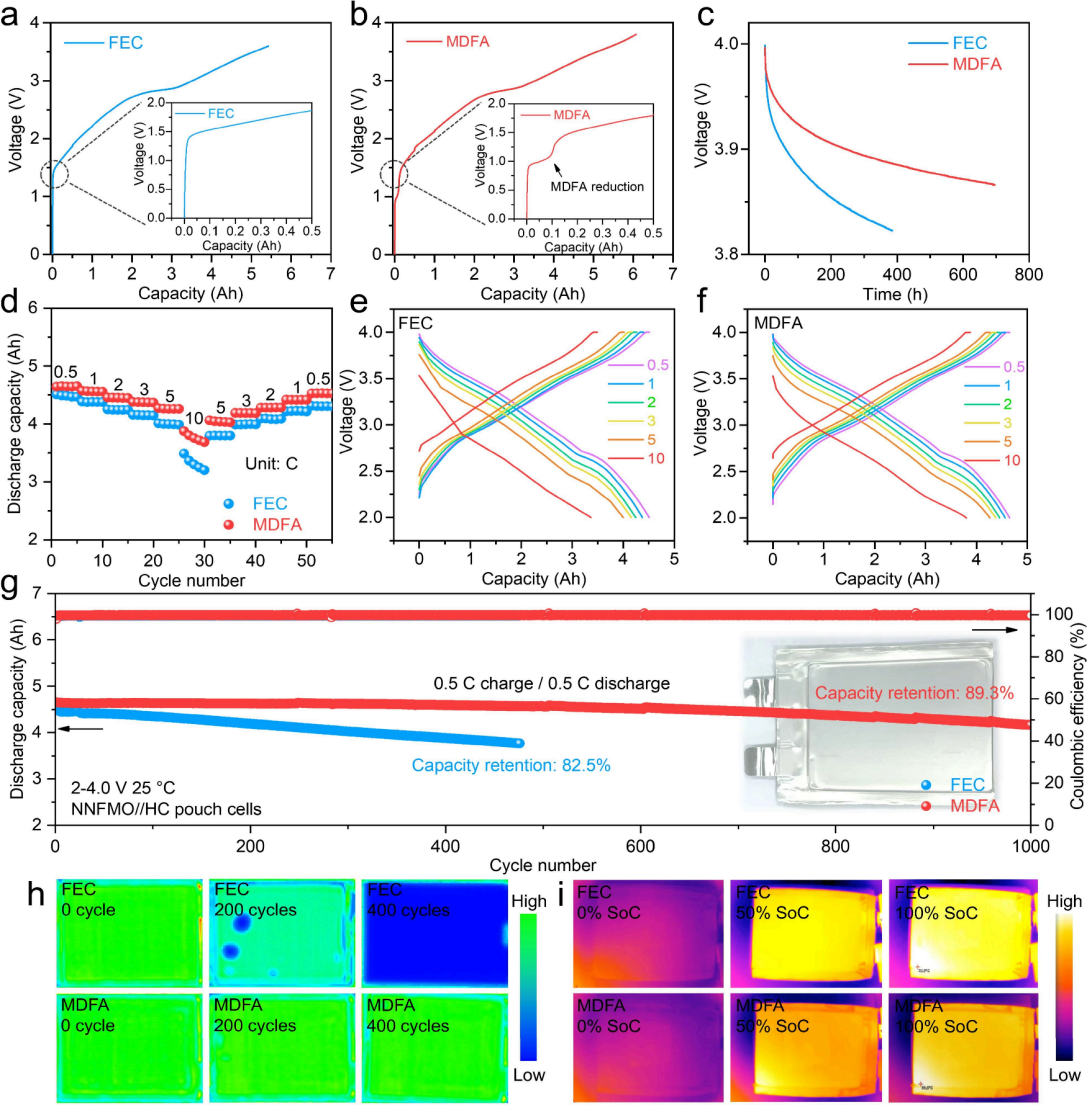
Electrochemical performance and characterizations of pouch cells. The initial formation curves of (a) FEC and (b) MDFA pouch cells. (c) Long-term storage ability comparison of pouch cells at room temperature. (d) Rate performance comparison of pouch cells and corresponding discharging/charging curves at different rates with (e) FEC and (f) MDFA additives. (g) Cycling performance comparison of pouch cells at 2–4 V and 25 °C. (h) Ultrasonic scanning images and (i) infrared images of pouch cells with FEC and MDFA addition.

Ultrasonic imaging was employed to visually monitor gas evolution within the pouch cells (Figure 5h). The ultrasonic signal was converted into a blue-green map, where green regions indicate adequate electrolyte infiltration and minimal gas formation and blue regions denote significant gas accumulation. Prior to cycling, both cells exhibited uniform green maps, suggesting good initial wetting. After 400 cycles, pronounced blue areas emerged in the FEC-based cell, reflecting severe gas generation, whereas the MDFA-based cell maintained a predominantly green appearance, indicating minimal gas evolution. Figure S39 shows the surface temperatures of pouch cells during 1 C discharging. Apparently, the surface temperature of the FEC pouch cell increases gradually to 27.6 °C. In contrast, the surface temperature of the MDFA pouch cell remains at 26.8 °C. Additionally, the surface temperature was tracked during the 2 C charging process. The FEC-based cell exhibited a higher temperature rise compared with the MDFA-based cell (Figure 5i), confirming that MDFA effectively suppresses internal side reactions in pouch cells.

## Conclusions

In conclusion, this work introduces a weak fluorine-bond electrolyte additive, MDFA, as a promising strategy to regulate SEI formation on HC anodes for SIBs. Owing to the electron-withdrawing O = S=O group, the S–F bond in MDFA is destabilized, enabling early decomposition and preferential SEI formation prior to Na^+^ adsorption. This leads to an inorganically rich SEI, which improves both ionic conductivity and mechanical stability. As a result, HC electrodes with MDFA exhibit good rate performance and long-term cycling stability. Notably, the assembled Ah-level pouch cell achieves 89.3% capacity retention after 1000 cycles, outperforming cells with conventional FEC additives. The suppression of side reactions also mitigates gas evolution and thermal diffusion, which contribute to improved thermal safety. This study provides a viable and scalable pathway for designing electrolyte additives tailored for high-performance sodium-ion pouch cells.

## Methods

### Raw Materials

EC (battery grade, DoDochem), PC (battery grade, DoDochem), DEC (battery grade, DoDochem), EMC (battery grade, DoDochem), FEC (battery grade, DoDochem), sodium hexafluorophosphate (NaPF_6_, battery grade, DoDochem), MDFA (98%, Macklin), layered oxides (NaNiFeMnO_2_, NNFMO, Canrd), HC (Kuraray), and sodium metal foil (Na, Changgao New Materials Co., Ltd.) were used without any treatment. All raw materials were stored in an argon-filled glovebox (oxygen < 0.1 ppm, water < 0.1 ppm).

### Electrode Preparation

All electrodes in pouch cells were produced by Zhuneng Tech. Co., Ltd. The NNFMO cathodes and HC anodes were prepared in a dry room (dew point ≤ −50 °C). The electrode preparation processes were similar in coin cells and pouch cells. A certain number of active materials (NNFMO or HC) were used to prepare Ah-level pouches of full cells. For cathode electrodes, 95.3 wt % NNFMO, 2.4 wt % conductive agent, and 2.3 wt % polyvinylidenefuoride were mixed in *N*-methyl-2-pyrrolidinone solvent to obtain the cathode slurry first. Then, the slurry was coated onto the aluminum foil using a transfer coater. The cathode electrodes were dried in an oven at 90 °C and cut into circular electrodes for coin cells or rectangular electrodes for pouch cells. For anode electrodes, 94.5 wt % HC, 2.0 wt % carbon black, 0.1 wt % CNTs, 1.2 wt % sodium carboxymethylcellulose, and 2.2 wt % styrene–butadiene rubber latex were mixed in deionized water obtained the anode slurry. The other preparation process of the anode electrodes was similar to that of the cathode electrodes.

### Battery Assembly

Single-faced electrodes were adopted for the coin cells. The coin cells used Na metal foil as the anode. The separator was a glass fiber. Thirty μL of electrolyte was added to each coin cell. The areal mass loading of HC electrodes is 3 mg cm^–2^ in coin cells. Pouch cells assembling: All electrodes were two-faced for pouch cells. The capacity of the pouch cells depends on the layers of electrodes. The cathode and anode electrodes were “Z-type” stacked. The cathode and anode tabs were aluminum and were welded by an ultrasonic bonding machine. The separator was a Celgard 2400 for pouch cells. The electrolyte amount was 4 g Ah^–1^ in each cell. After the electrolytes were added, the pouch cells were aged in an oven at 45 °C for 3 days. More parameters of pouch cells are displayed in the following table. All cells were tested in a Neware battery cycler (MIHW-200-160CH-B, Shenzhen, China).The FEC electrolyte was 1 M NaPF_6_ in the EC/PC/EMC/DEC mixed solvent (1/1/1/1 volume ratio) with 1 wt % FEC as the additive. The MDFA electrolyte was obtained by adding 1 wt % MDFA to replace FEC.

### Characterizations

The morphology of materials was observed by field emission SEM (Nova Nano SEM 450) and field emission TEM (Tecnai G2 F30). The crystal structure of the samples was analyzed by the X-ray diffraction (Bruker) spectrum with an Al Ka radiation (*h*v = 1486.6 eV) beam. The interfacial components of the electrodes after cycling were analyzed by XPS (AXIS-Ultra DLD-600W) and TOF-SIMS (ION-TOF GmbH, lon tof Gmhb 5). The standard spectrum of C 1s was 284.6 eV. The Ar^+^ ion beam was adopted to etch the surface.

### Electrochemical Measurements

The electrochemical windows of the electrolytes were measured simultaneously according to linear scan voltammetry technology: stainless steel as the working electrode, Na metal as the counter electrode, and reference electrodes. The voltage scanning speed and range were 1 mV s^–1^ and 0–2 V, respectively. The ionic conductivity (σ) of electrolytes was tested with a conductometer. The ionic conductivity was calculated using the following equation:

$$\sigma =\frac{L}{S\times R}$$

where *L* is the distance between the two working electrodes, *S* is the area of the working electrode, and *R* is the resistance of the system.

The ultrasonic images of pouch cells were obtained in an ultrasonic scanner (Topsound Technology Co. Ltd., China). The temperature of the pouch cells during cycling was monitored by an infrared camera (Suzhou Keyiqian Precision Equipment Co., Ltd.). The pouch cell was preformatted in a battery formation test equipment (MP-68-G-128, Chenwei Co. Ltd.). During preformation, the applied pressure and temperature were 0.2 MPa and 35 °C, respectively. Then, the gas pockets of pouch cells were removed, and the pouch cells were packed finally. All pouch cells were fixed by two aluminum plateaus and cycled in the Neware battery cycler with a voltage window of 2–4 V. The pouch cell was cycled in a constant temperature oven at 25 °C except for additional declaration.

### Computational Methods

All theoretical calculations were carried out using Materials Studio software. First, all molecules were geometrically optimized. The function theory was local-density approximation and piecewise continuous. The basis set was DNP. The maximum force, the maximal atomic displacement, and the self-consistent field convergence are <0.002 Ha/Å, <5 × 10^–3^ Å, and <10^–5^ eV, respectively. The binding energy (*E*_binding_) between Na^+^ and molecules was calculated according to the following equation:

$$E_{\mathrm{binding}}=E_{{\mathrm{Na}}^{+}-\mathrm{molecule}}-E_{\mathrm{molecule}}-E_{{\mathrm{Na}}^{+}}$$

*E*_Na^+^–molecule_, *E*_molecule_, and *E*_Na^+^_ correspond to the binding energy of the Na^+^-molecule cluster, molecule, and Na^+^, respectively.

Molecular dynamics (MD) simulations of the FEC and MDFA electrolytes were conducted on the Forcite module. The FEC electrolyte snapshot contained 40 Na^+^, 40 PF_6_^–^, 150 EC, 114 PC, 91 EMC, 94 DEC, and 5 FEC. The MDFA electrolyte snapshot contained 40 Na+, 40 PF6-, 150 EC, 114 PC, 91 EMC, 94 DEC, and 5 MDFA. The force field was COMPASSII. The long-range electrostatic interactions of electrolytes were adopted by the Ewald method. All of the snapshots were simulated at the NPH pattern with a coupling constant of 200 ps. After the volume of snapshots remained stable, the simulations were carried out at the NVT pattern for 1 ns until the equilibrated state. The time step of all of the simulations was 1 fs. The coordination number (*n*) of A particles (anions, solvents) around the Na^+^ was calculated through the radial distribution function [RDF, *g*(*r*)] according to the following equation:

$$n_{{\mathrm{Na}}^{+}-A}(r)=\int_{0}^{r}4\pi r^{2}\rho_{\mathrm{molecule}}g_{{\mathrm{Na}}^{+}-A}(r)dr$$

where ρ_*A*_ is the average number density of the molecule or anion in the bulk electrolyte. *r* is the distance between Na^+^ and the molecule.

## Supplementary Material



## Notes

Molecular structures, bond lengths, binding energies, ELFs, MD simulations, SEM images, TEM images, GITT analysis, CV curves, charge/discharge voltage curves, capacity ratios, postcycle analysis, XPS analysis, performance comparison, and energy density (DOCX)
