# Supplementary material for: A Weak-Fluorine-Bond Molecule Stabilizes Hard Carbon Anodes for Practical Sodium-Ion Batteries
Source: ACS Nano. 2025 Aug 16;19(33):30466–75. doi: 10.1021/acsnano.5c10983 (PMC12755197; doi:10.1021/acsnano.5c10983)
Supplement: Supplementary file 1 [file nn5c10983_si_001.docx]

Supporting Information

**A Weak-Fluorine-Bond Molecule Stabilizes Hard Carbon Anodes for Practical Sodium-Ion Batteries**

Yaqi Liao†,∆, Han Liu†,∆, Yangqian Zhang†, Jiayi Yang†,* Haijin Ji‡, Donghai Wang₴,* Lixia Yuan‡, Yunhui Huang‡, and Yang Ren†,§,*

† Department of Physics, JC STEM Lab of Energy and Materials Physics, City University of Hong Kong, Hong Kong 999077, P. R. China

‡ State Key Laboratory of Material Processing and Die and Mold Technology, School of Materials Science and Engineering, Huazhong University of Science and Technology, Wuhan, 430074, China

₴ School of Renewable Energy, Hohai University, Nanjing 211100, China

§ Shenzhen Research Institute, City University of Hong Kong, Shenzhen 518057, P. R. China

* Corresponding author

∆ These authors contributed equally to this work

E-mail addresses: jiayyang@cityu.edu.hk (J.Y. Yang); wangdonghai@hhu.edu.cn (D.H. Wang), yangren@cityu.edu.hk (Y. Ren)


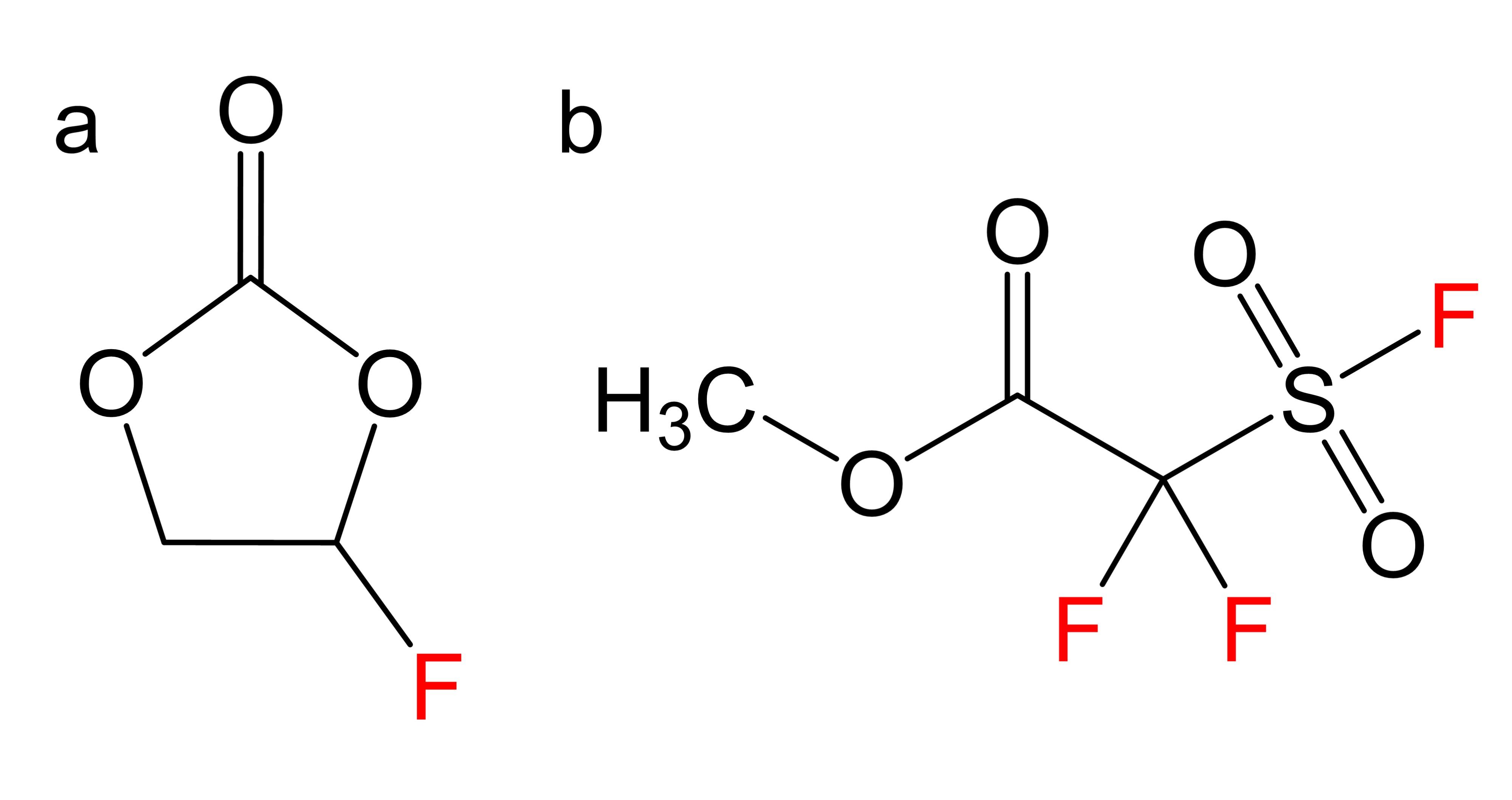


**Figure S1.** The molecular structures of (a) FEC and (b) MDFA.


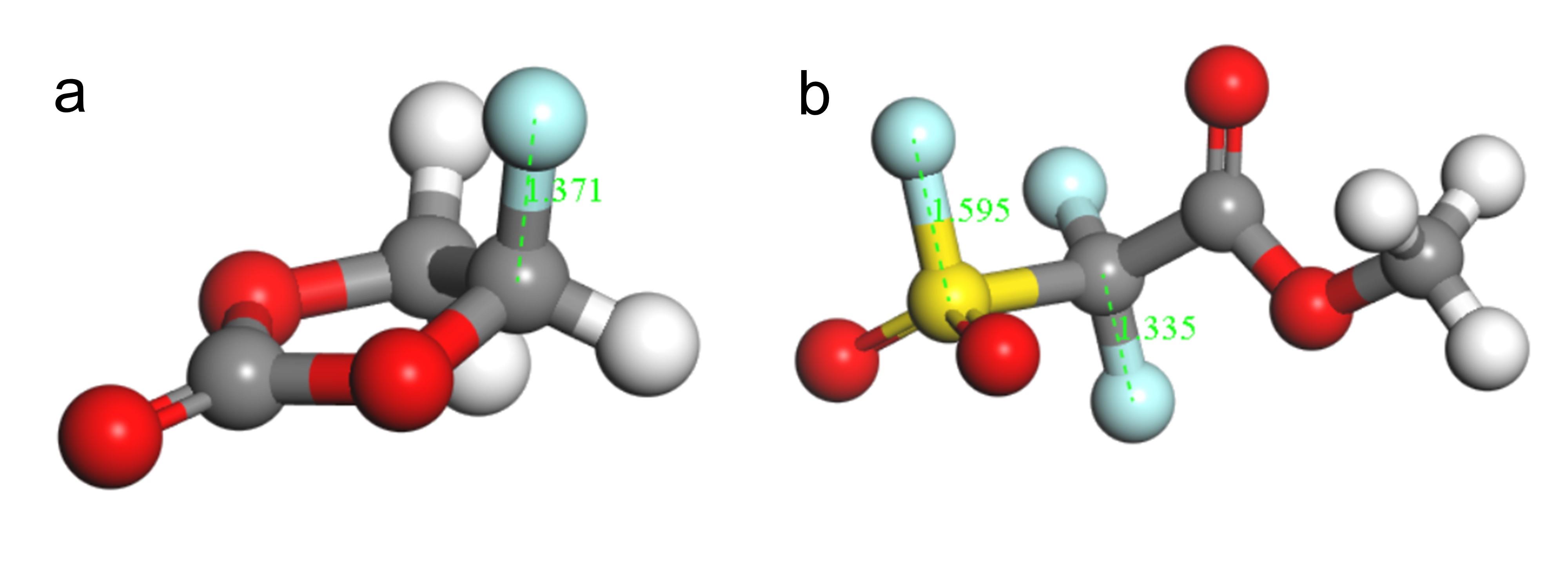


**Figure S2.** The fluorine-bond lengths of (a) FEC and (b) MDFA molecules.


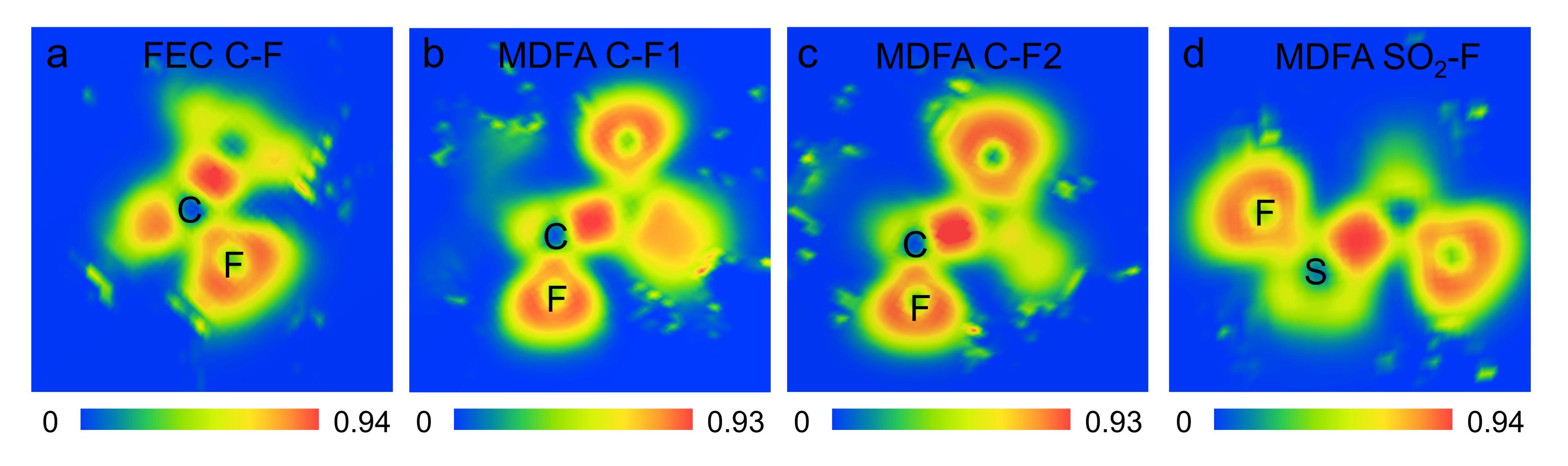


**Figure S3.** The electron location function of the (a) C−F bond of FEC, (b) C−F1, (c) C−F2, and (d) SO_2_−F bonds of MDFA.


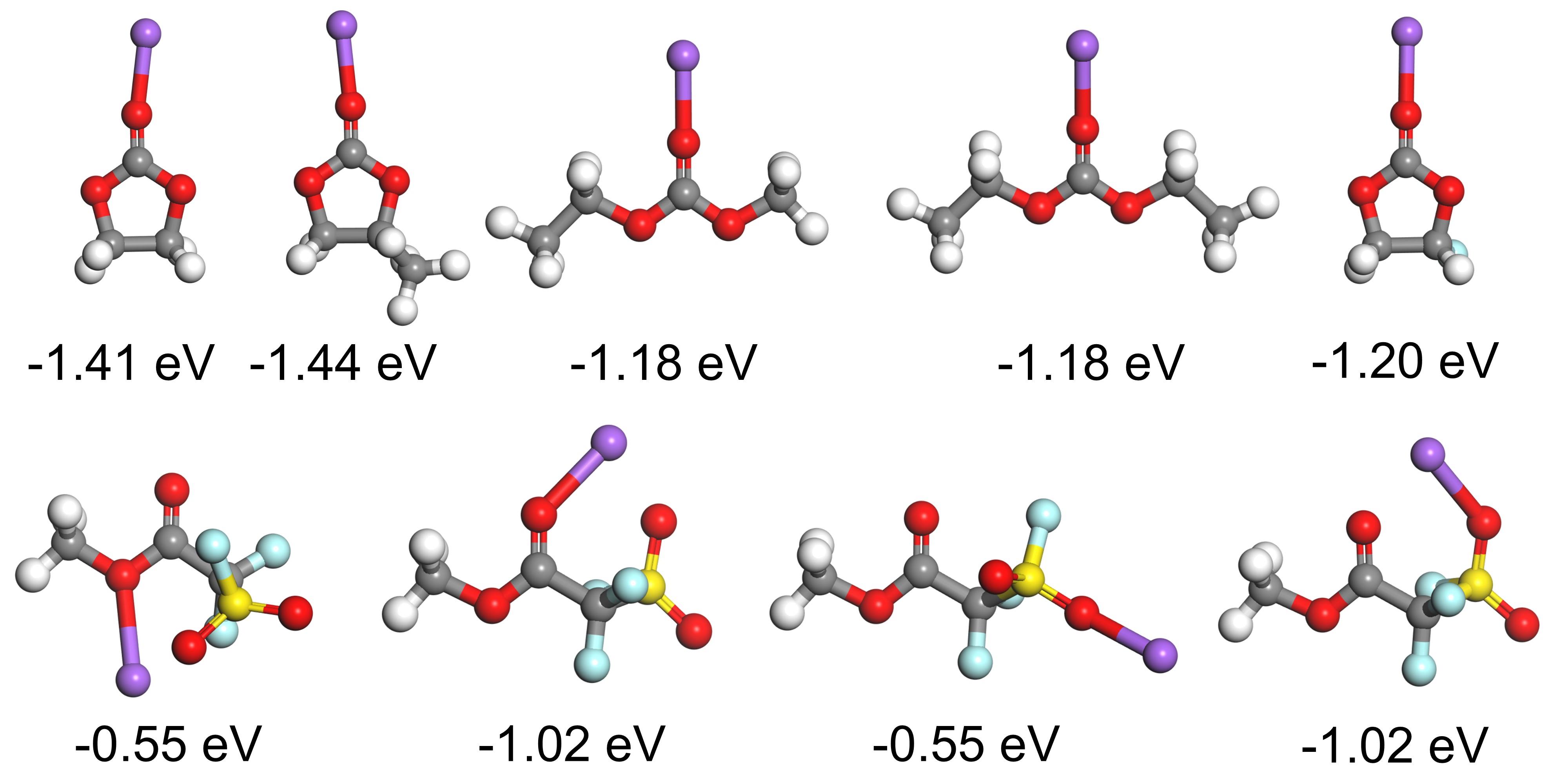


**Figure S4.** The binding energies of Na^+^ and different molecules.


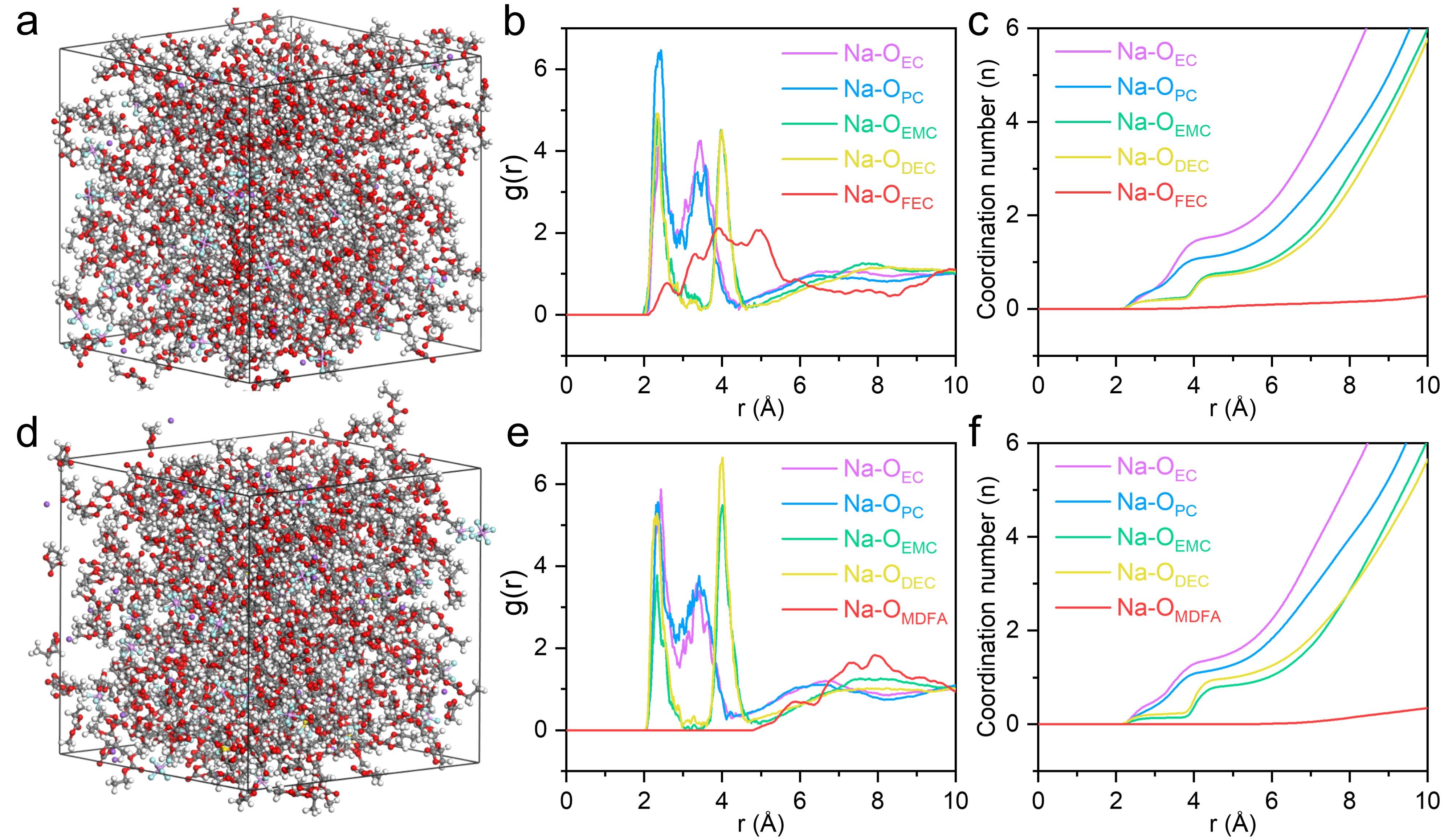


**Figure S5.** Molecular dynamics simulations of bulk electrolytes. Snapshots of (a) FEC and (d) MDFA electrolytes. Radial distribution function of (b) FEC and (e) MDFA electrolytes. Coordination number analysis of (c) FEC and (f) MDFA electrolytes.


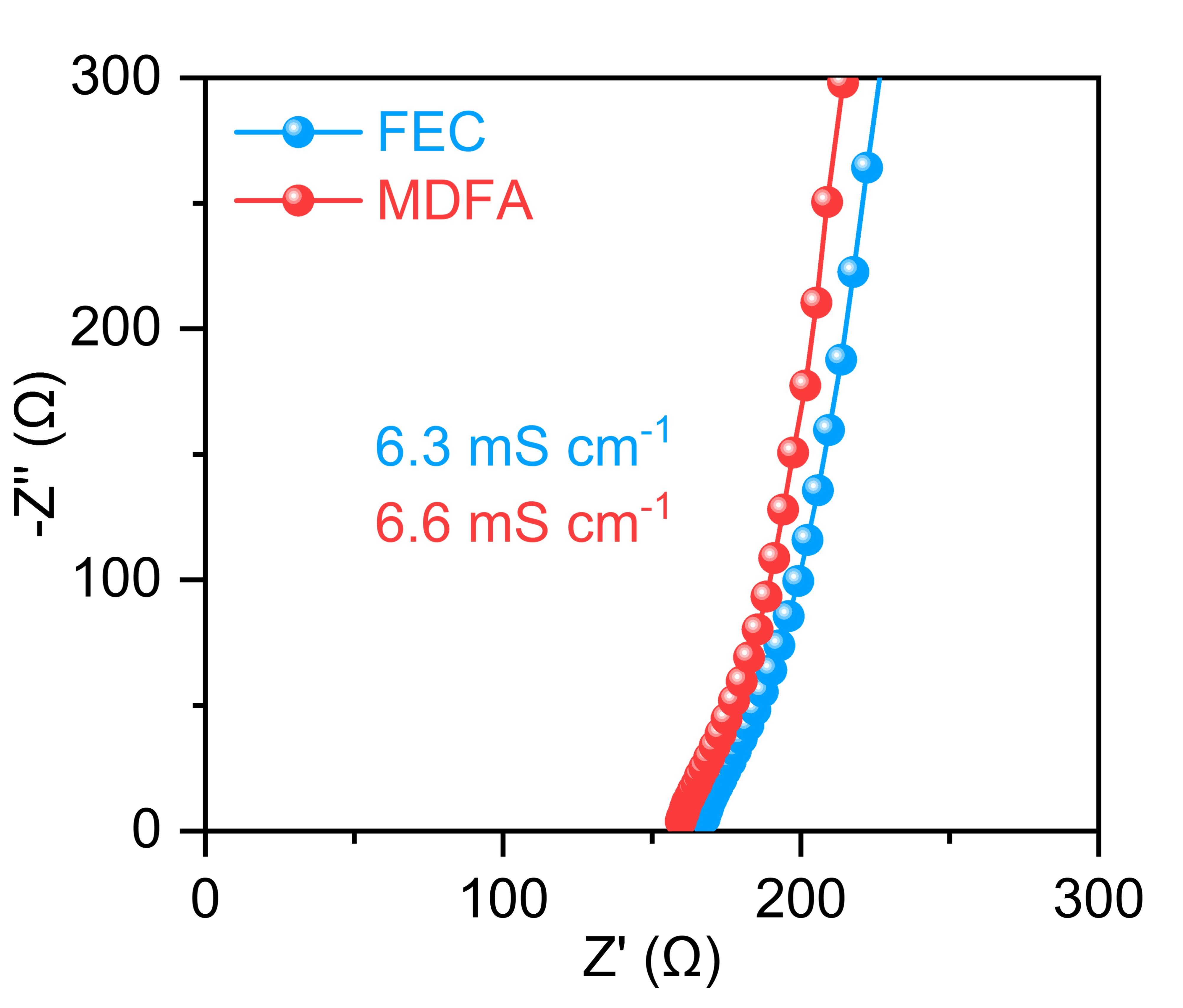


**Figure S6.** The ionic conductivities of the FEC and MDFA electrolyte.


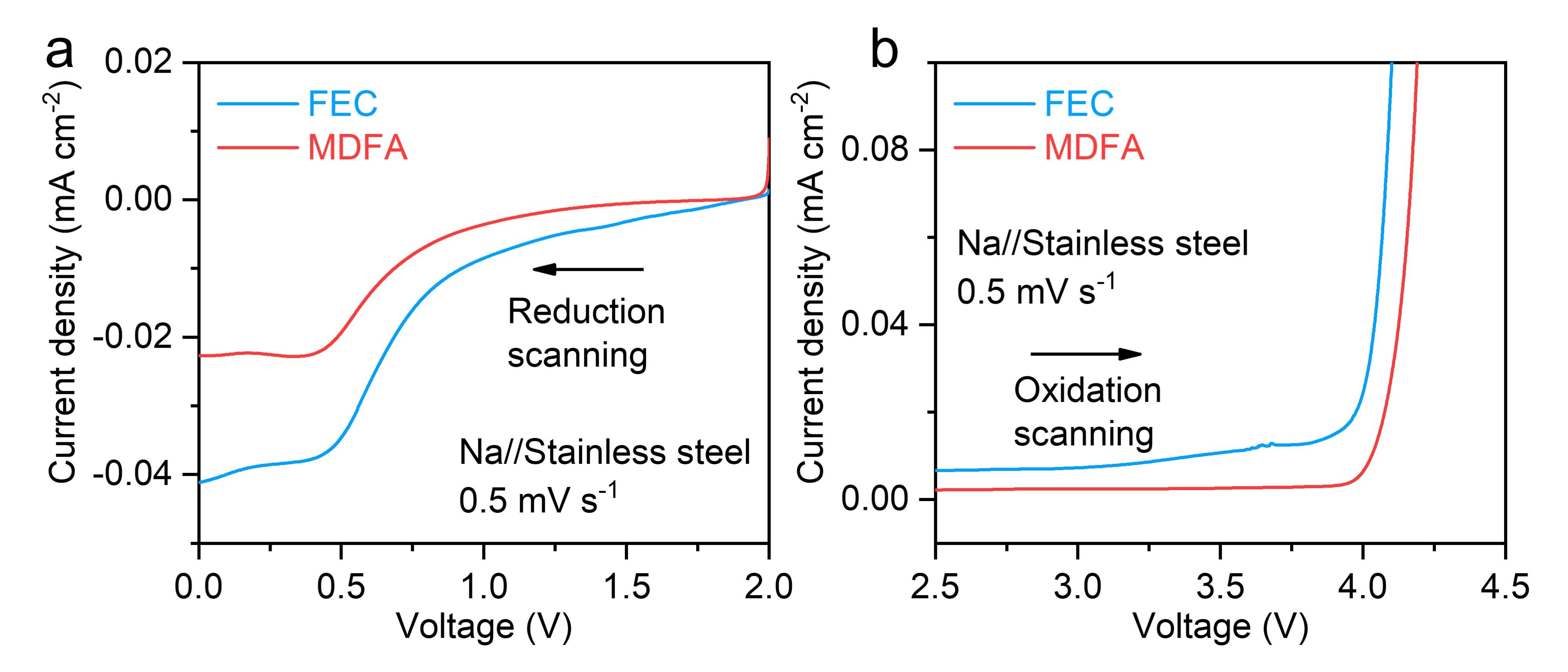


**Figure S7.** The electrochemical window of electrolytes at (a) reduction and (b) oxidation process.


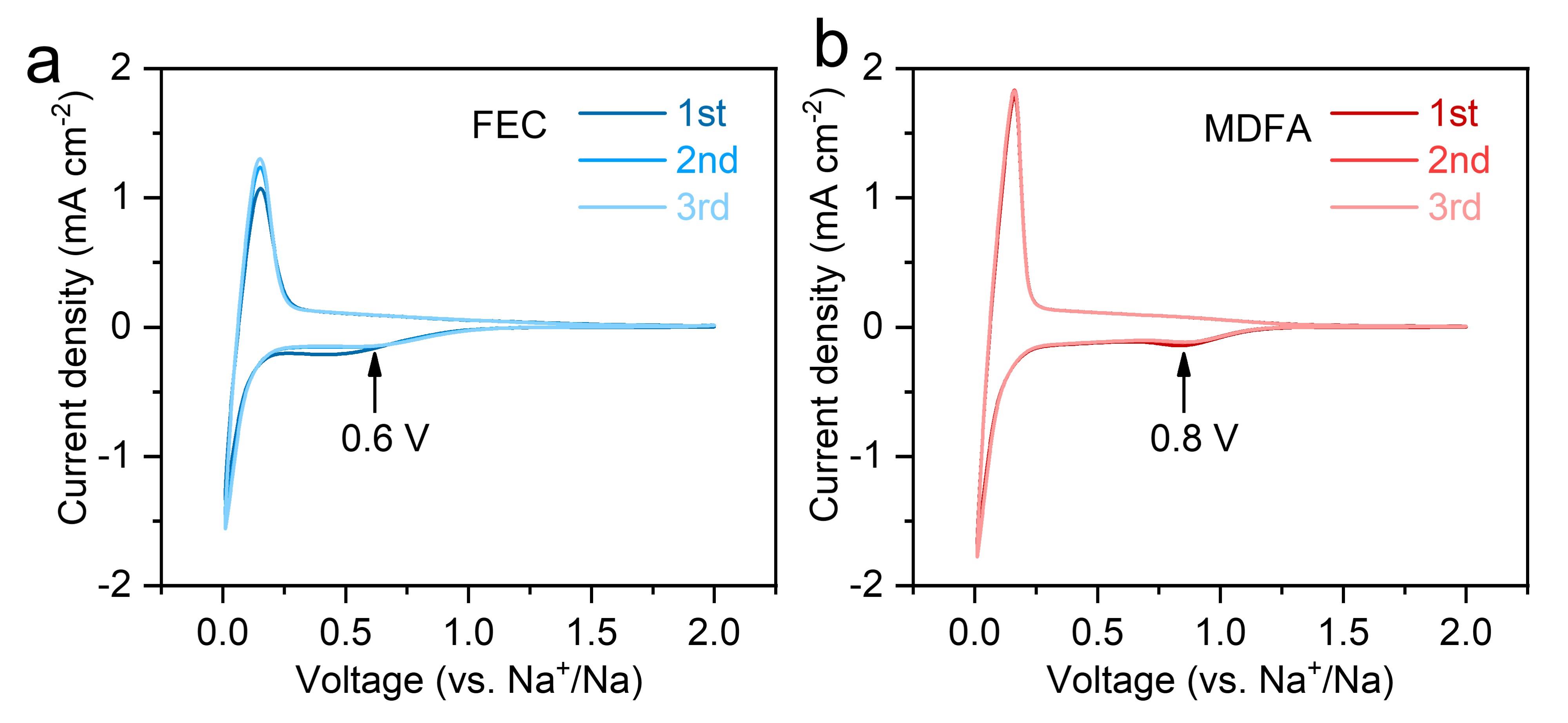


**Figure S8.** The cyclic voltammogram curves of the (a) FEC and (b) MDFA electrolytes during three cycles.


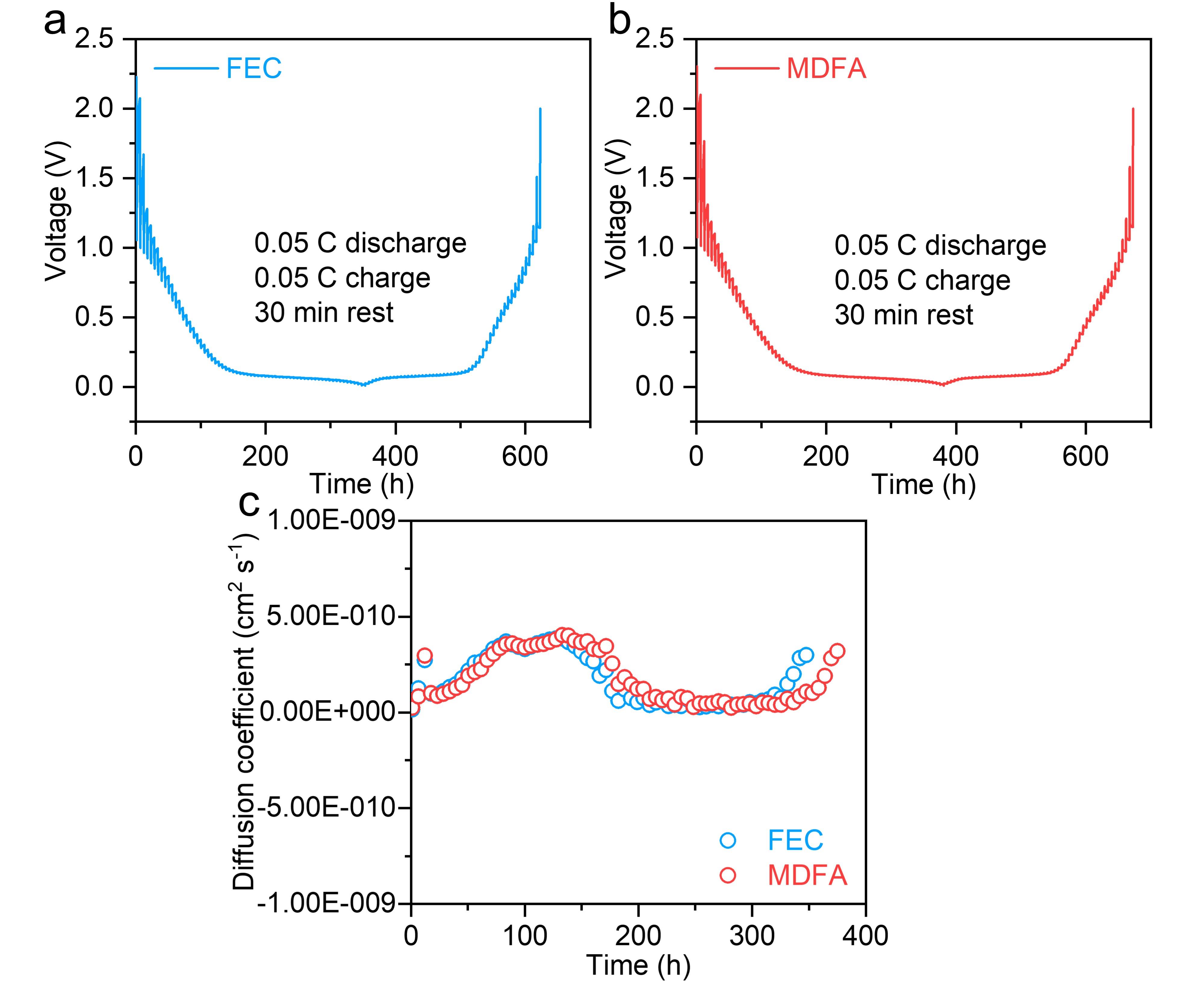


**Figure S9.** The galvanostatic intermittent titration test of cells: the time-voltage curve of the (a) FEC and (b) MDFA cells. (c) The Na^+^ diffusion coefficient.


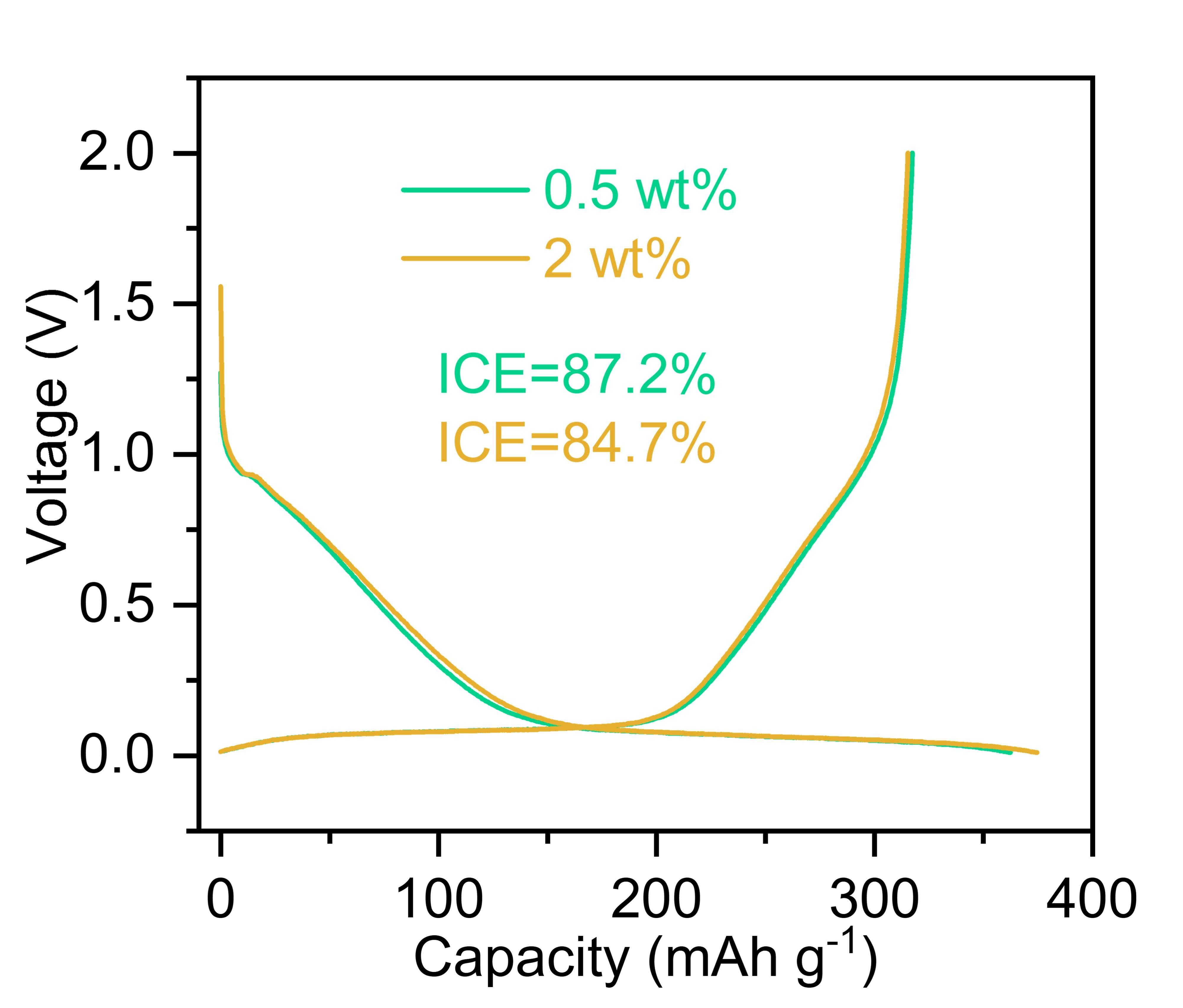


**Figure S10.** The initial discharging/charging curve of cells with 0.5 and 2 wt% MDFA.


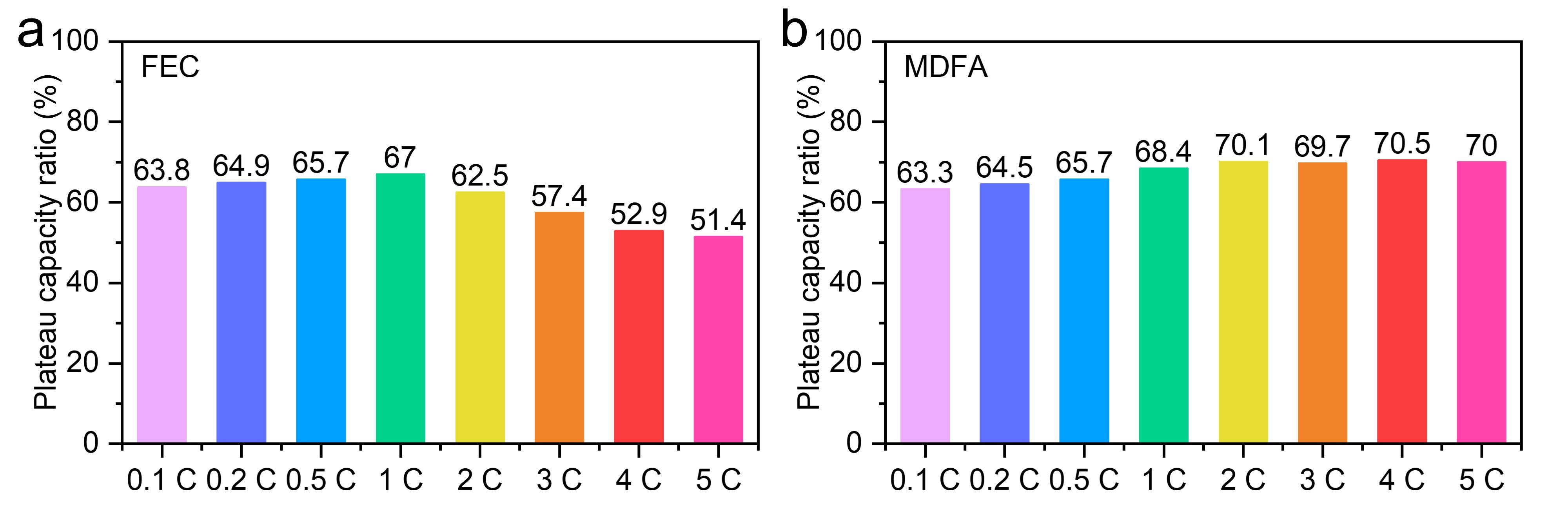


**Figure S11.** The plateau capacity ratios in the (a) FEC and (b) MDFA cells.


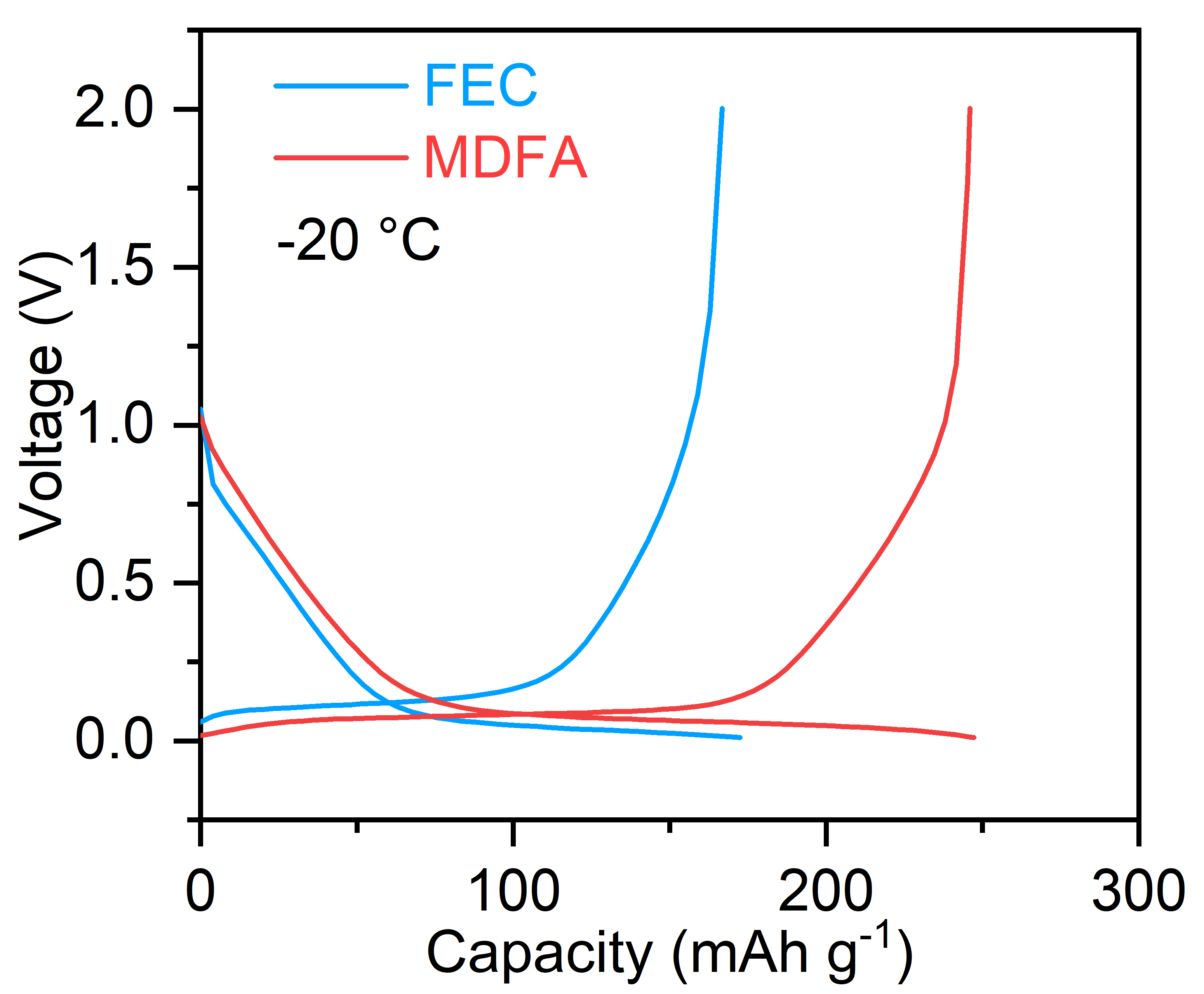


**Figure S12.** The discharging/charging curves of cells at −20 °C and 0.5 C.


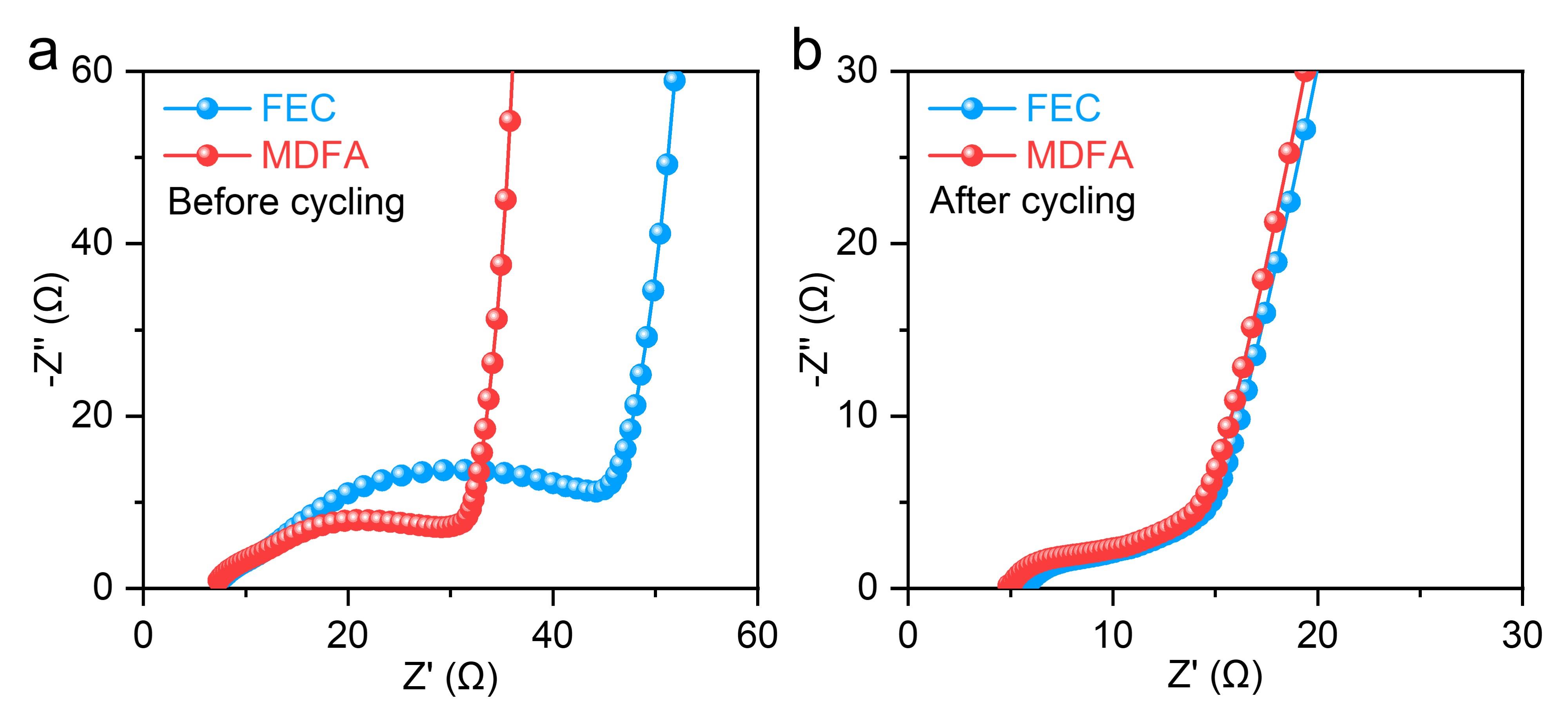


**Figure S13.** The electrochemical impedances (a) before and (b) after cycling.


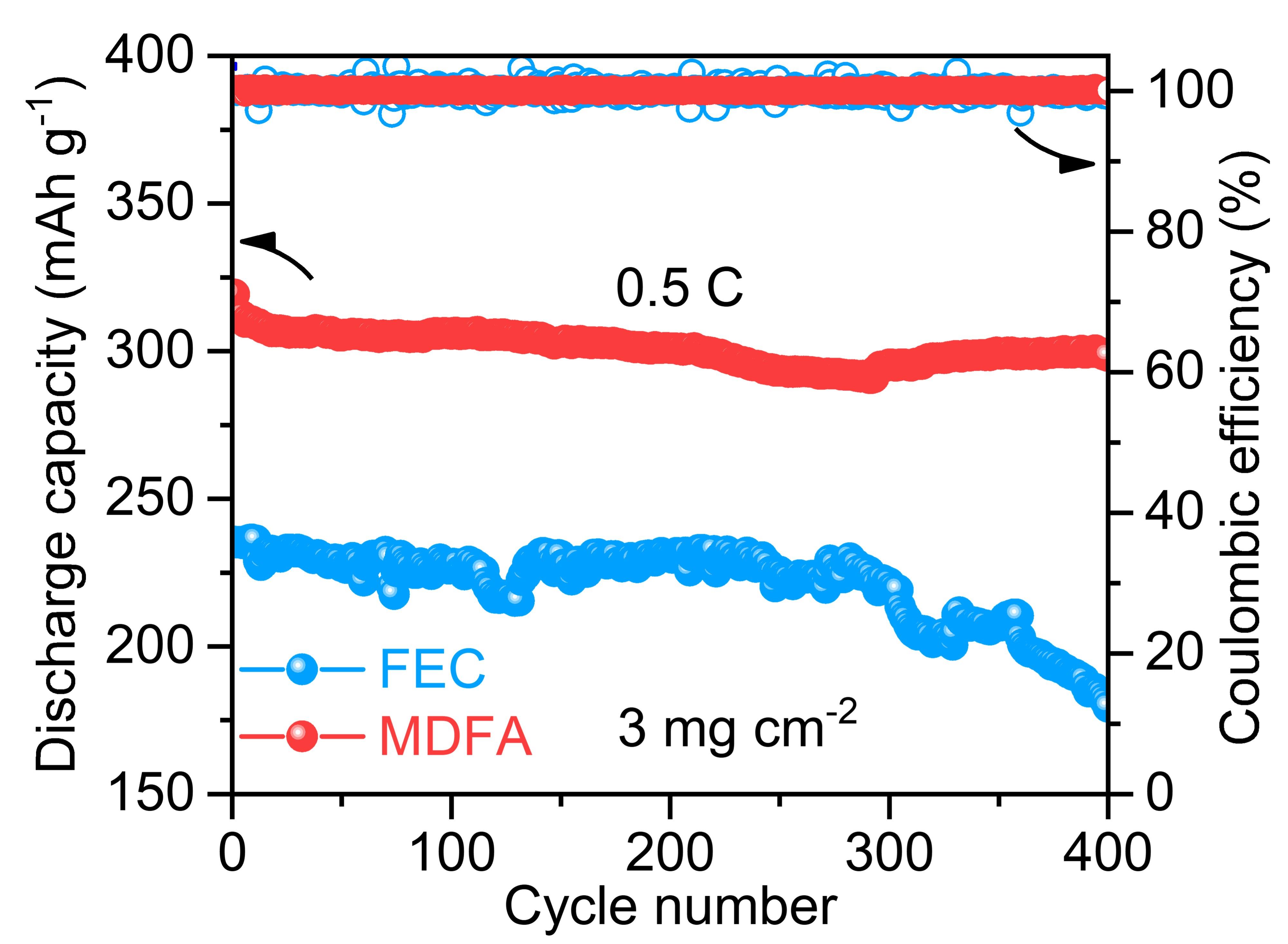


**Figure S14.** The cycling stability of the FEC and MDFA cells at 0.5 C.


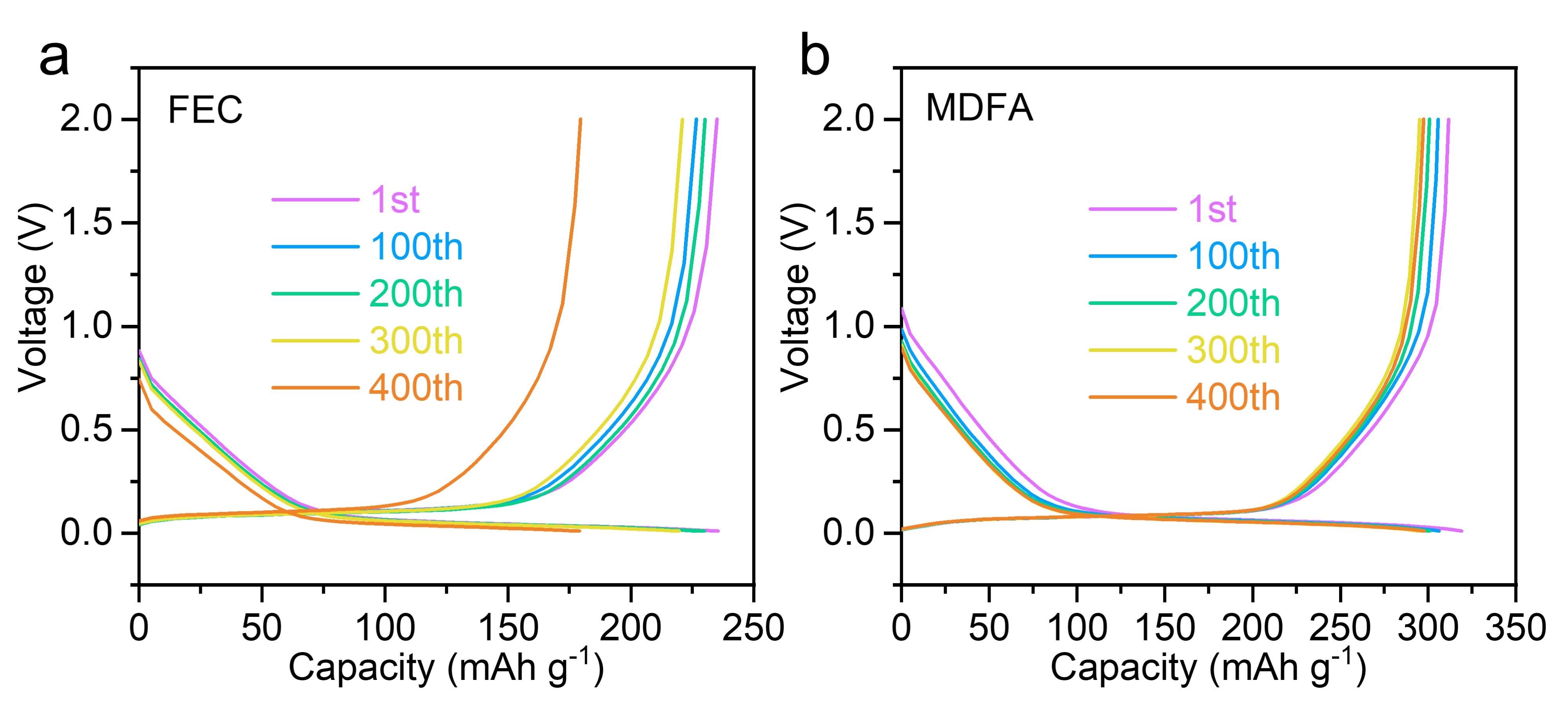


**Figure S15.** The discharging/charging curves of the (a) FEC and (b) MDFA cell at 0.5 C.


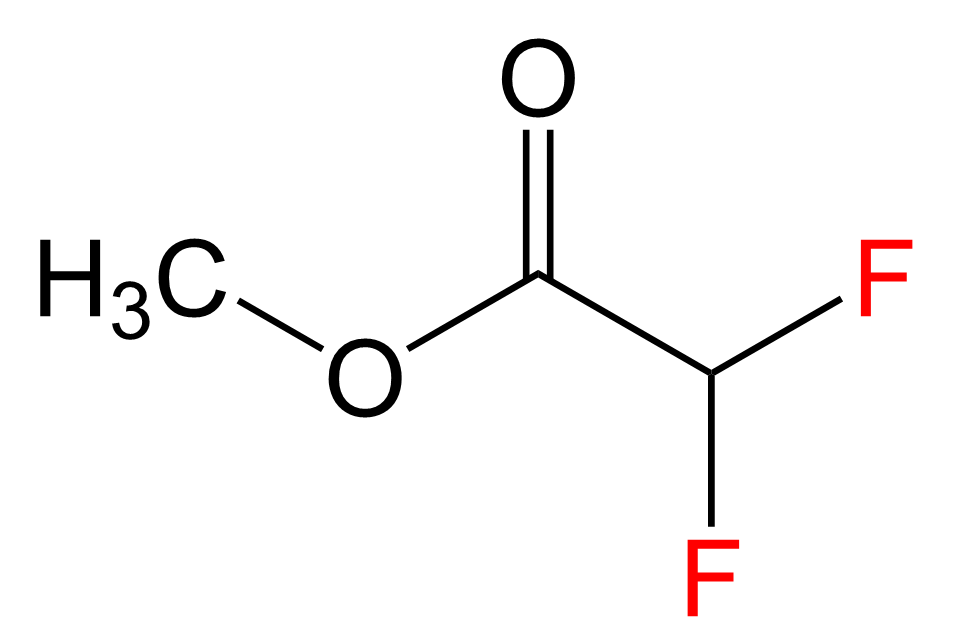


**Figure S16.** The molecular structure of methyl difluoroacetate (MFA).


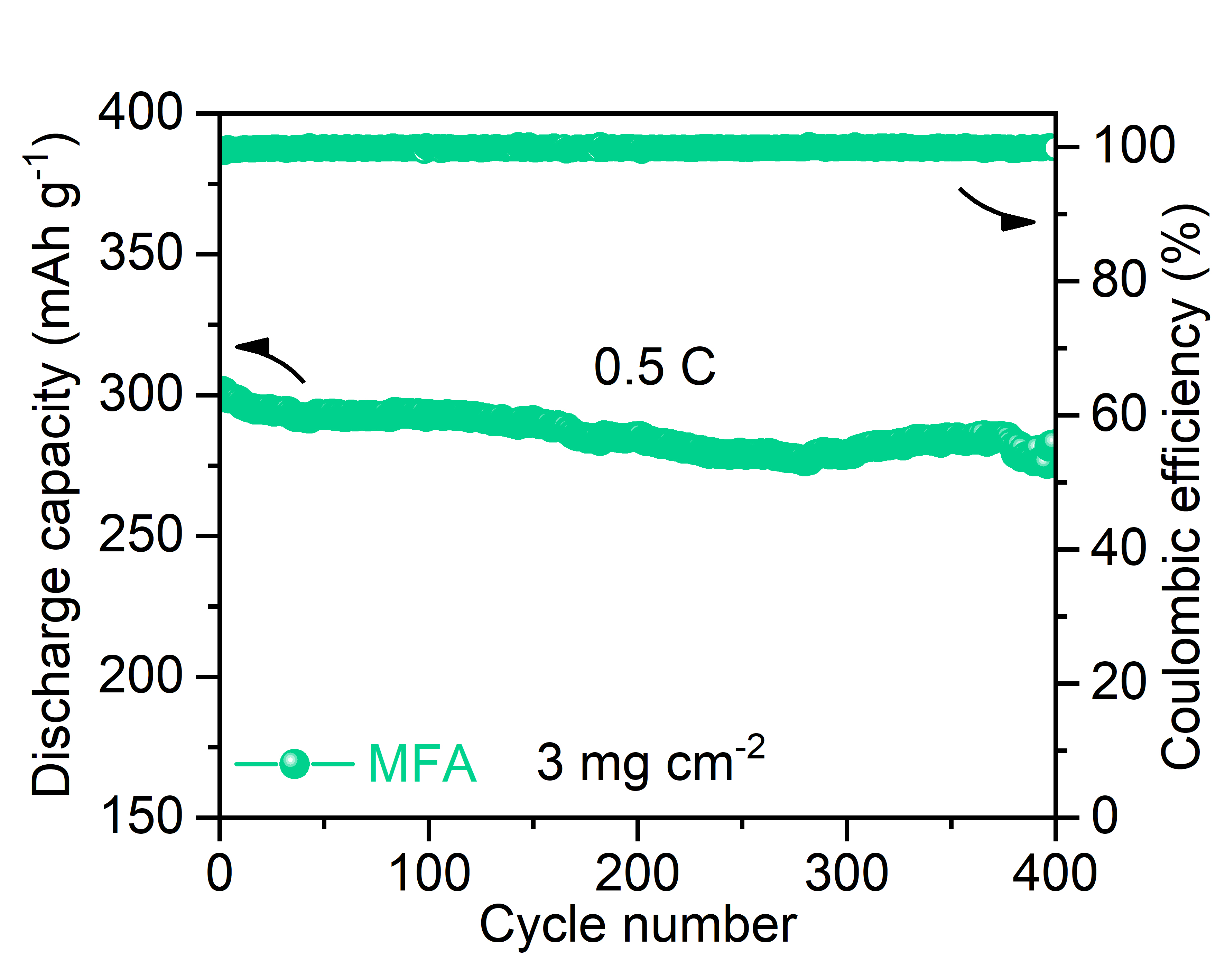


**Figure S17.** The cycling performance of the cell with the MFA additive.


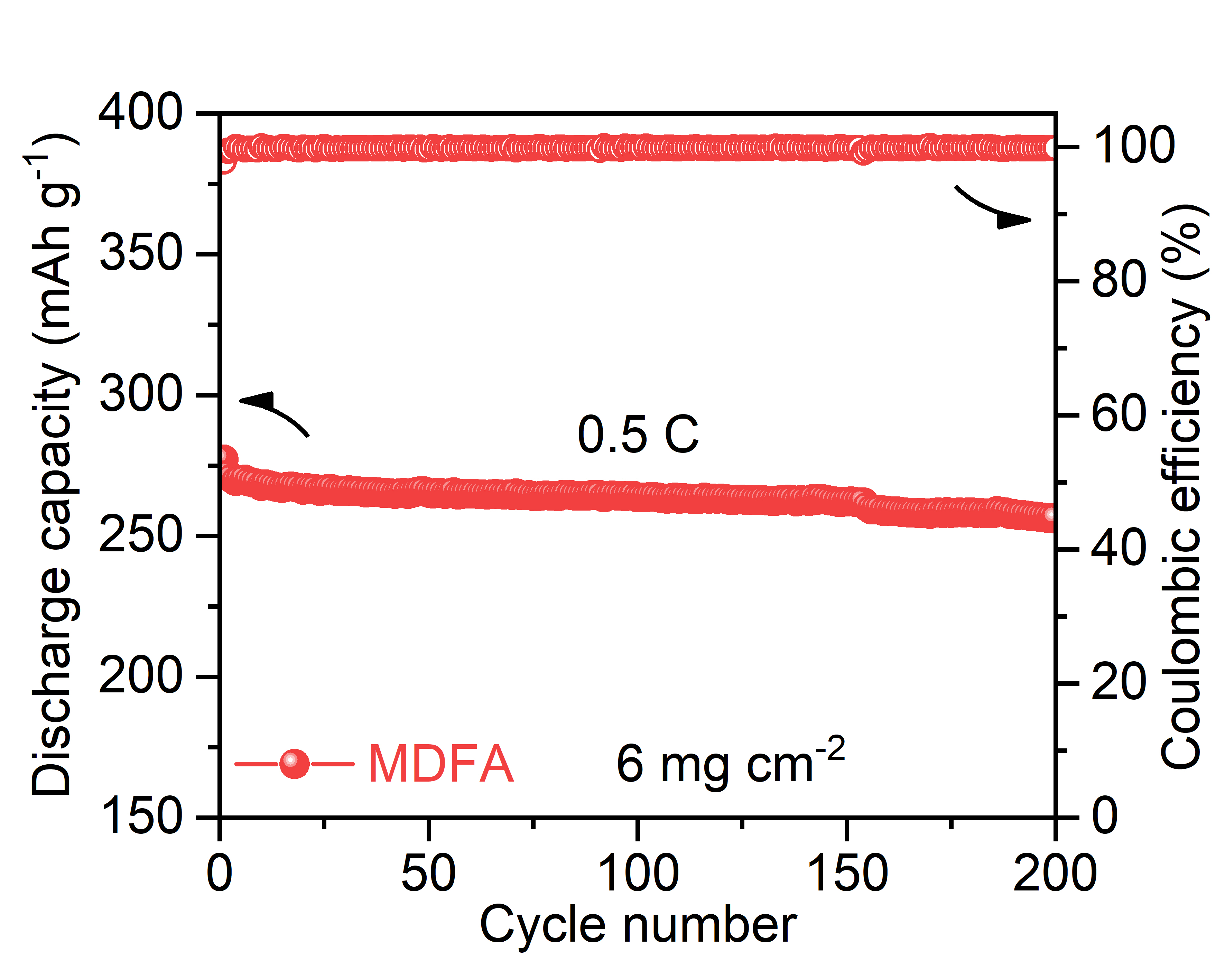


**Figure S18.** The cycling performance of the MDFA cell at 6 mg cm^−2^.=


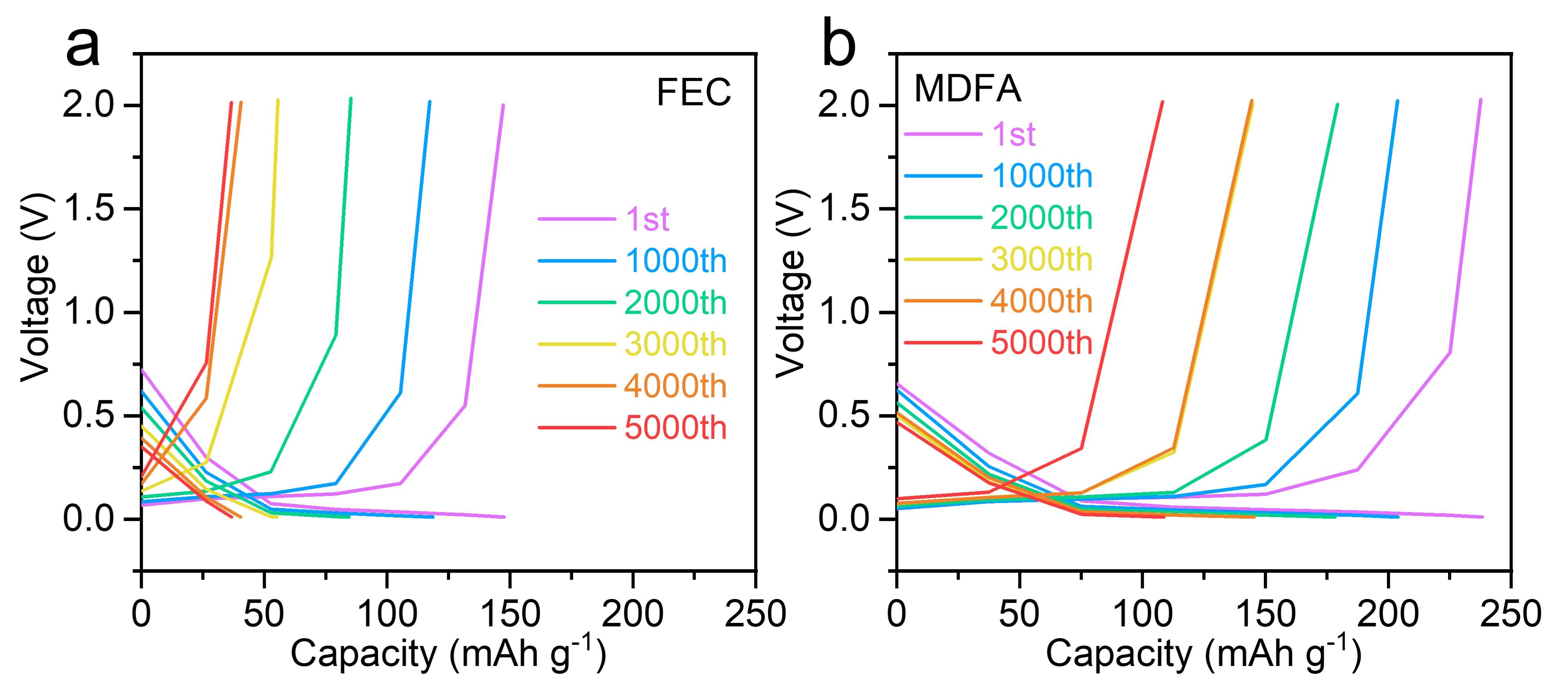


**Figure S19.** Cell discharging/charging curves in the (a) FEC and (b) MDFA electrolytes.


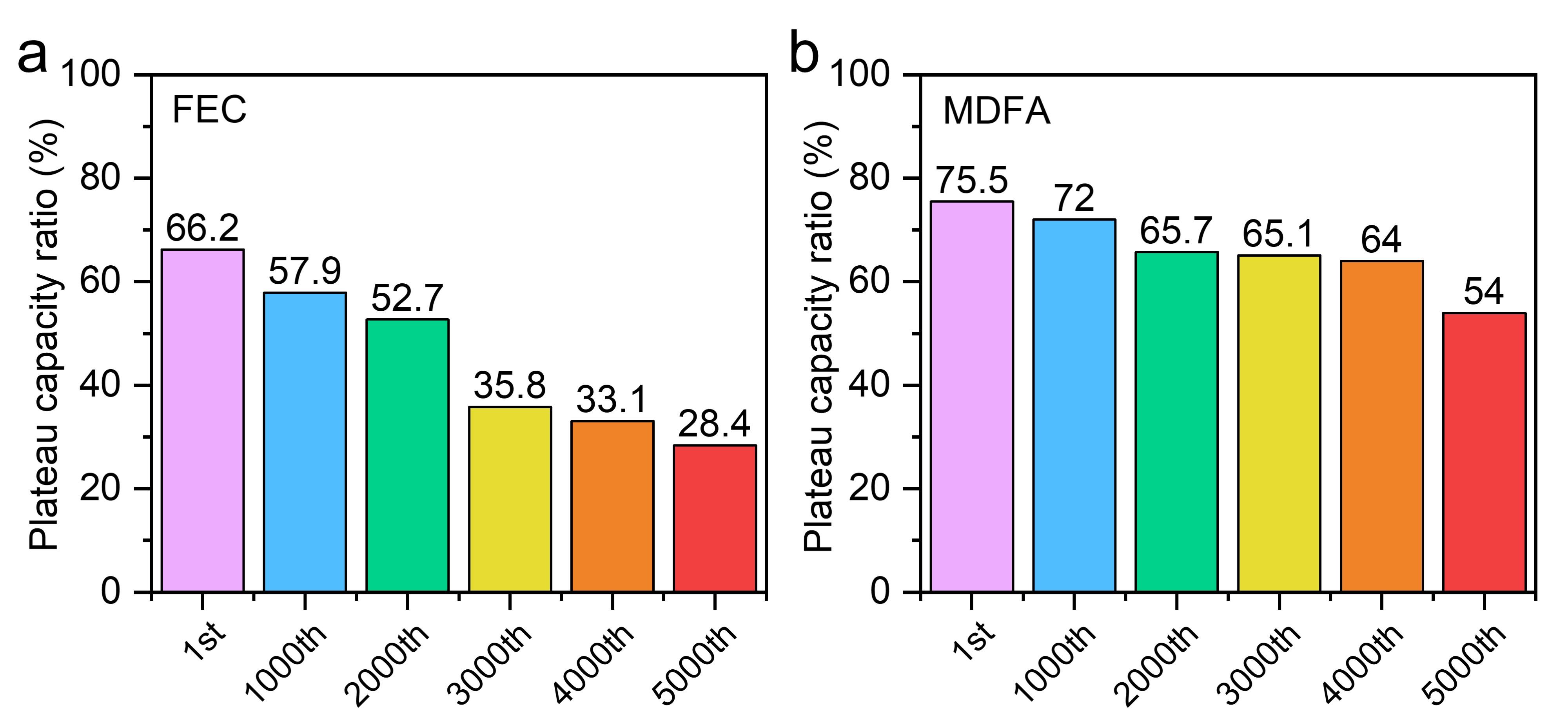


**Figure S20.** The plateau capacity ratios of the hard carbon during long cycling.


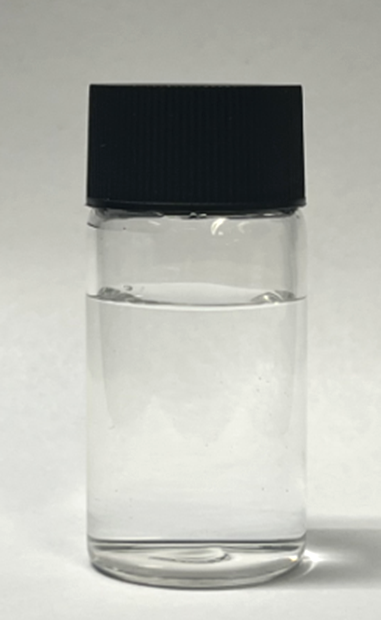


**Figure S21.** The digital photo of the electrolyte with MDFA after one-week aging.

**
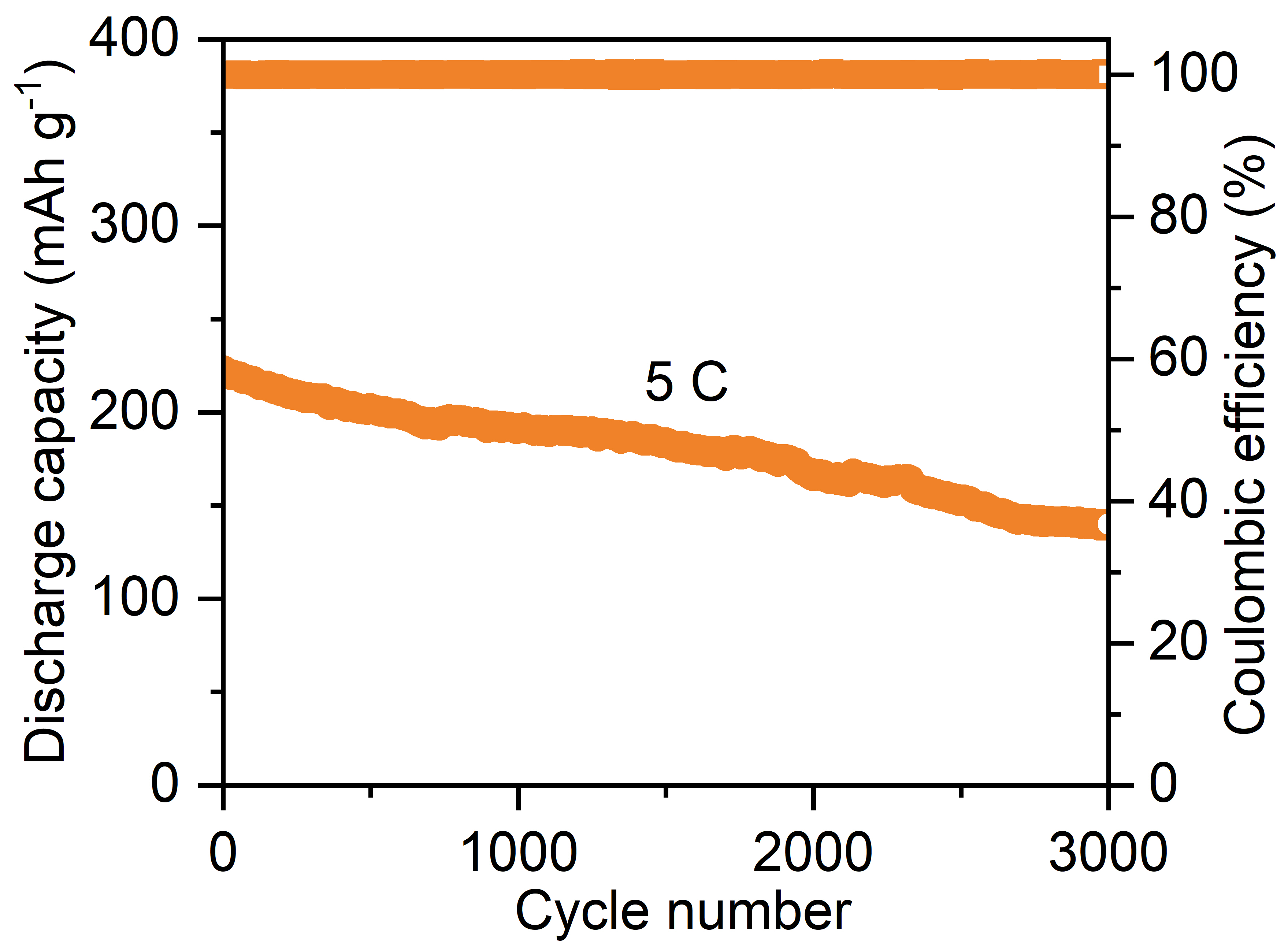
**

**Figure S22.** The cycling performance of the cell with the one-week aging MDFA electrolyte.


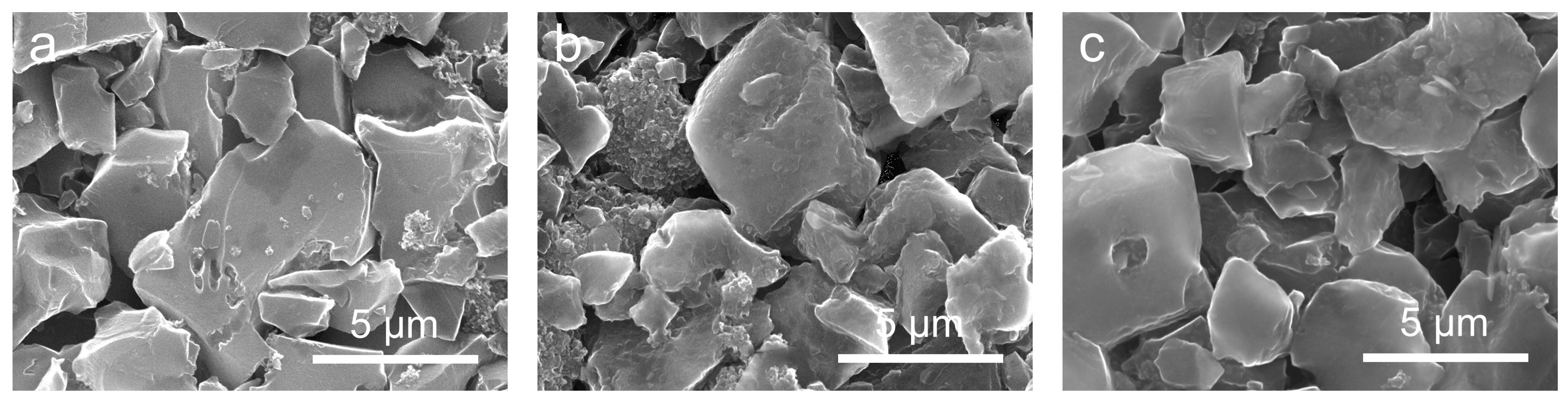


**Figure S23.** The morphology of HC electrodes (a) before cycling and after cycling in the (b) FEC and (c) MDFA electrolytes.


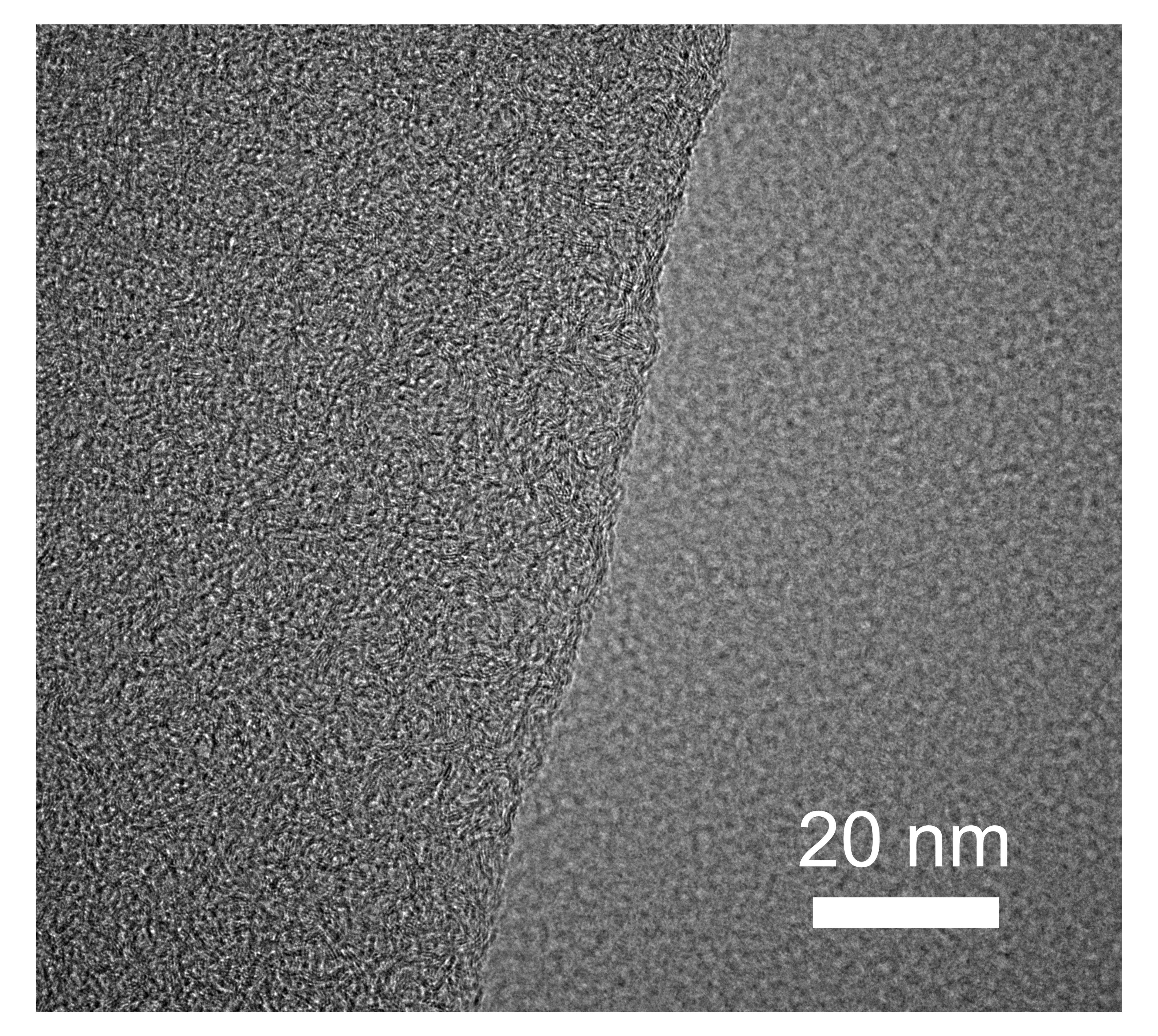


**Figure S24.** The TEM image of HC before cycling.


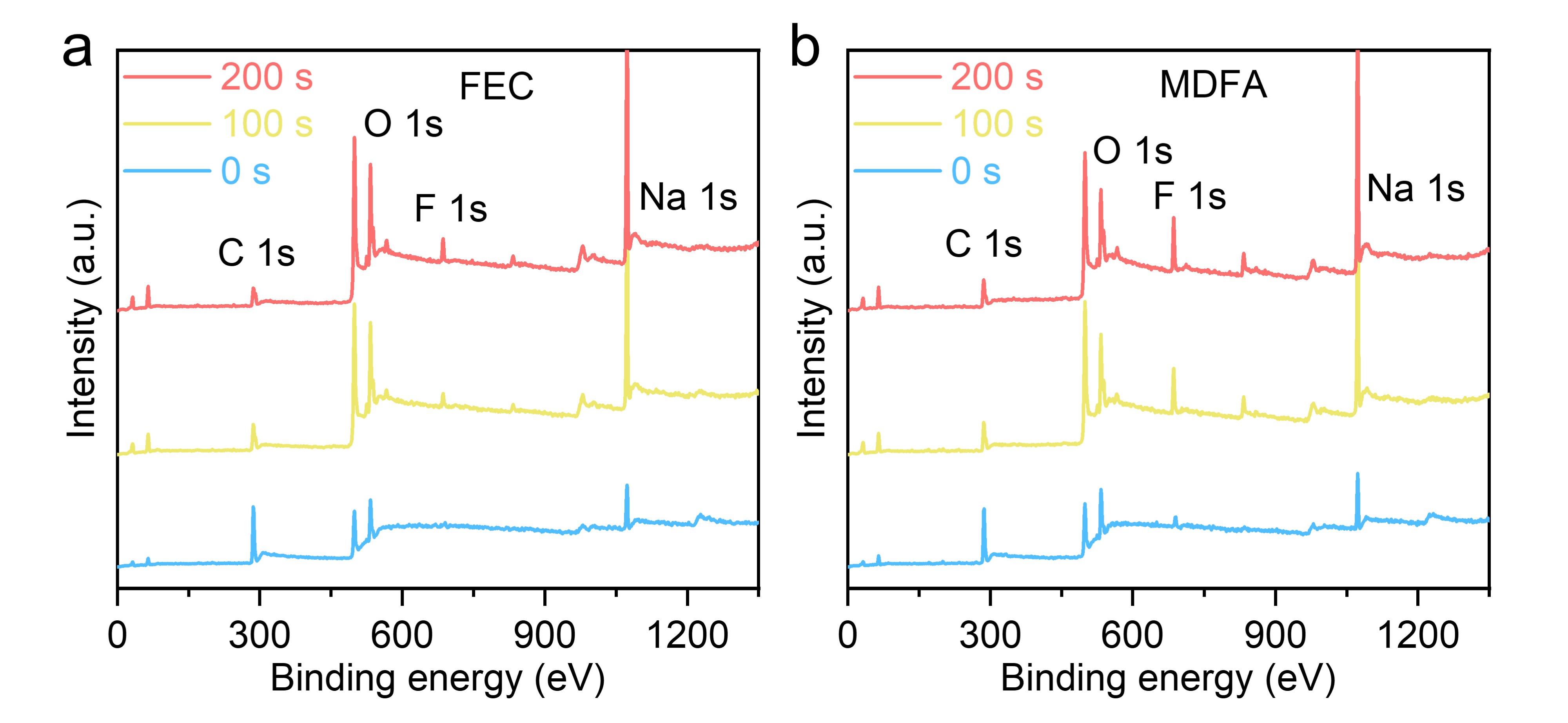


**Figure S25.** The broad XPS spectra in the (a) FEC and (b) MDFA electrolytes.


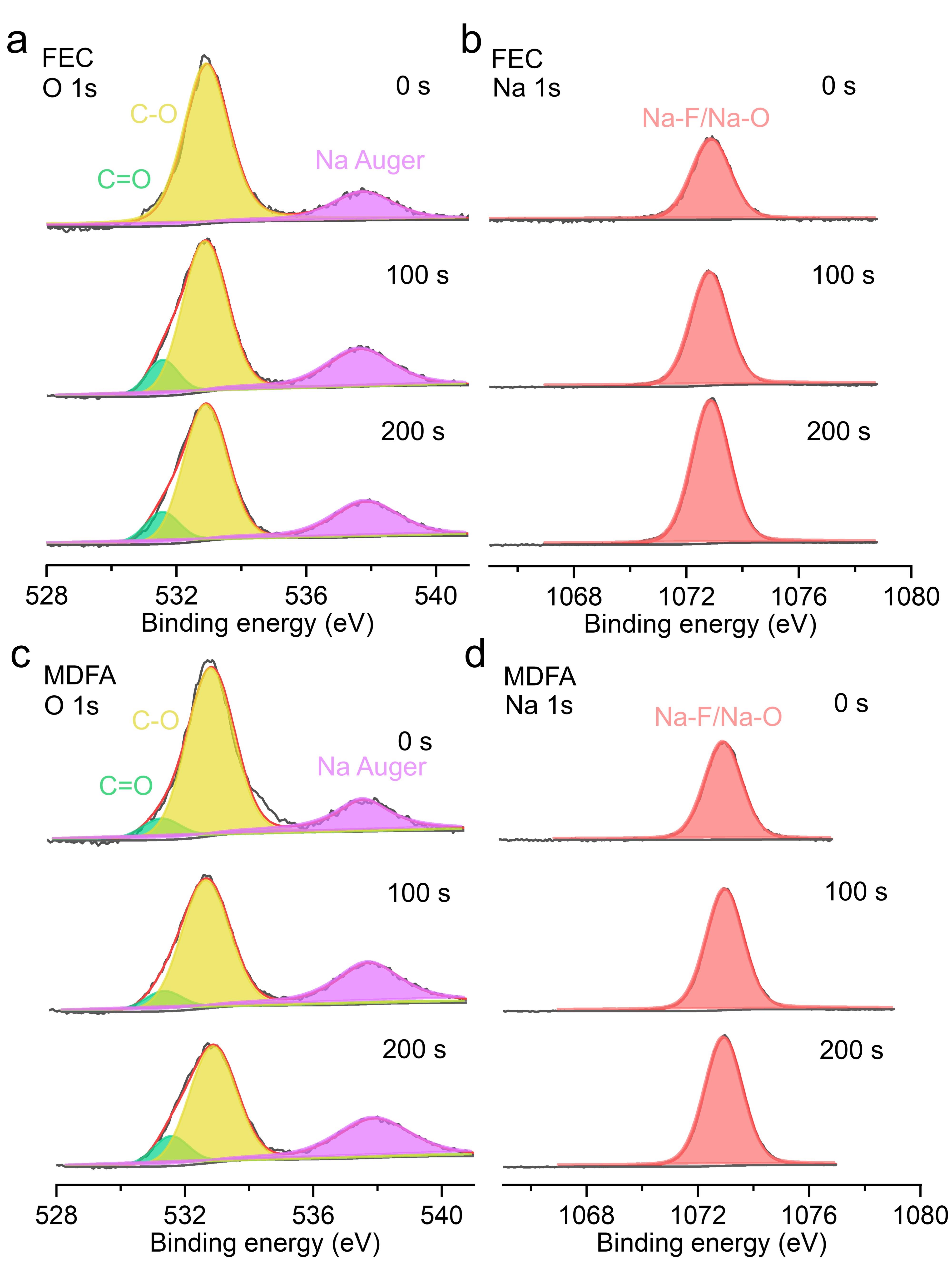


**Figure S26.** The (a) O 1s and (b) Na 1s spectra in the FEC electrolyte. The (c) O 1s and (d) Na 1s spectra in the MDFA electrolytes.


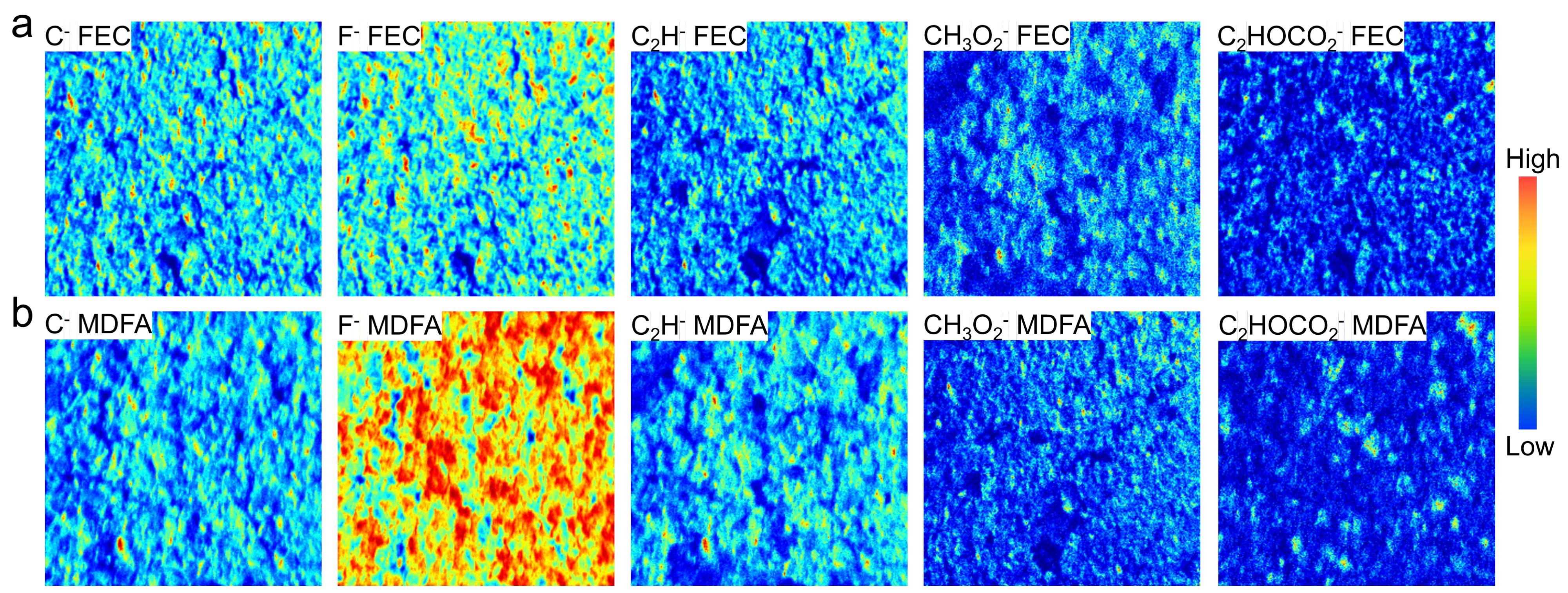


**Figure S27.** The fragment contents at the XY plateau are in (a) FEC and (b) MDFA electrolytes.


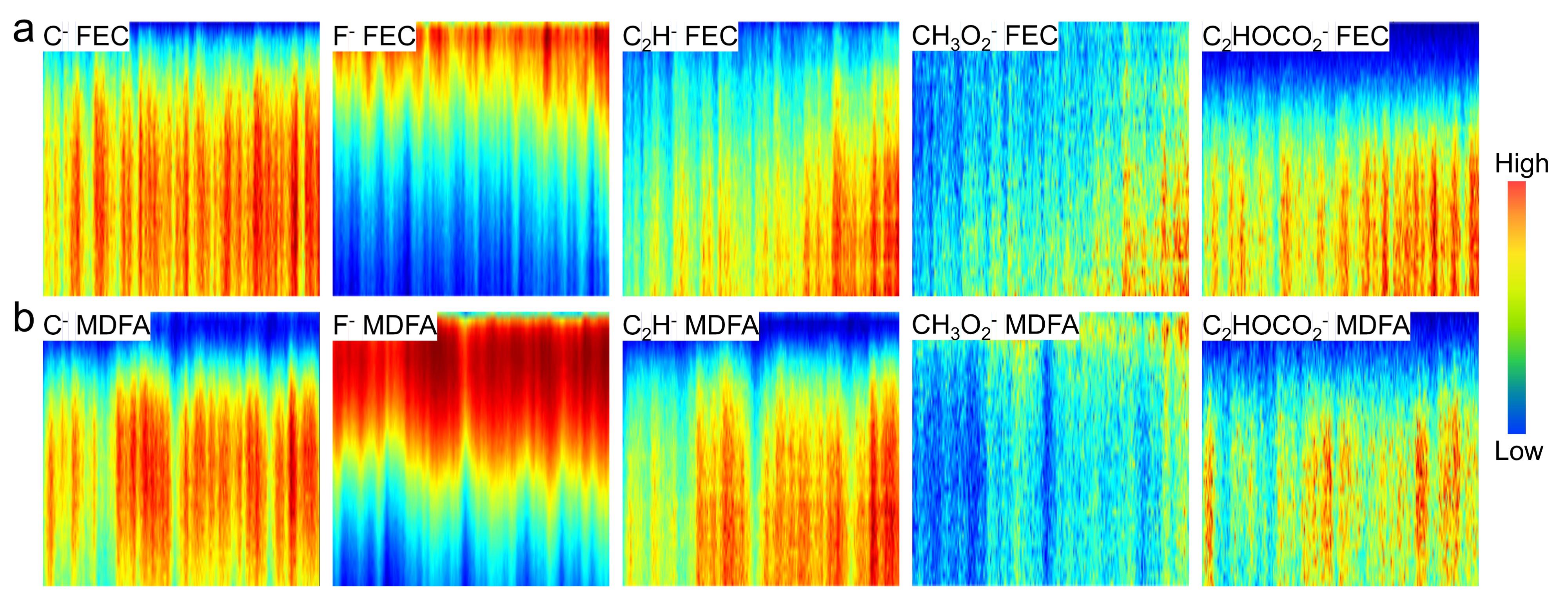


**Figure S28.** The fragment contents at the XZ plateau are in (a) FEC and (b) MDFA electrolytes.


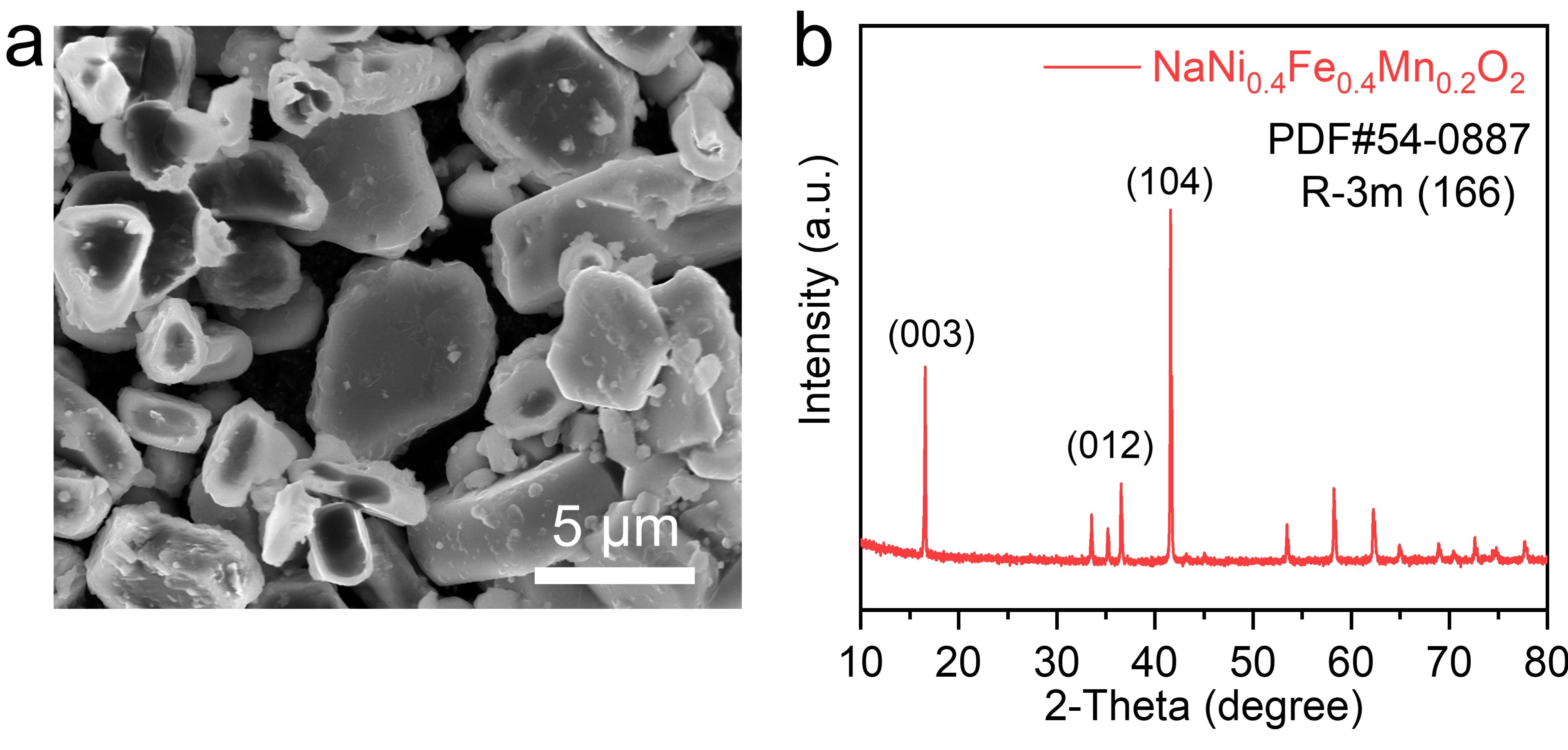


**Figure S29.** The (a) morphology and (b) XRD spectrum of the NNFMO material.


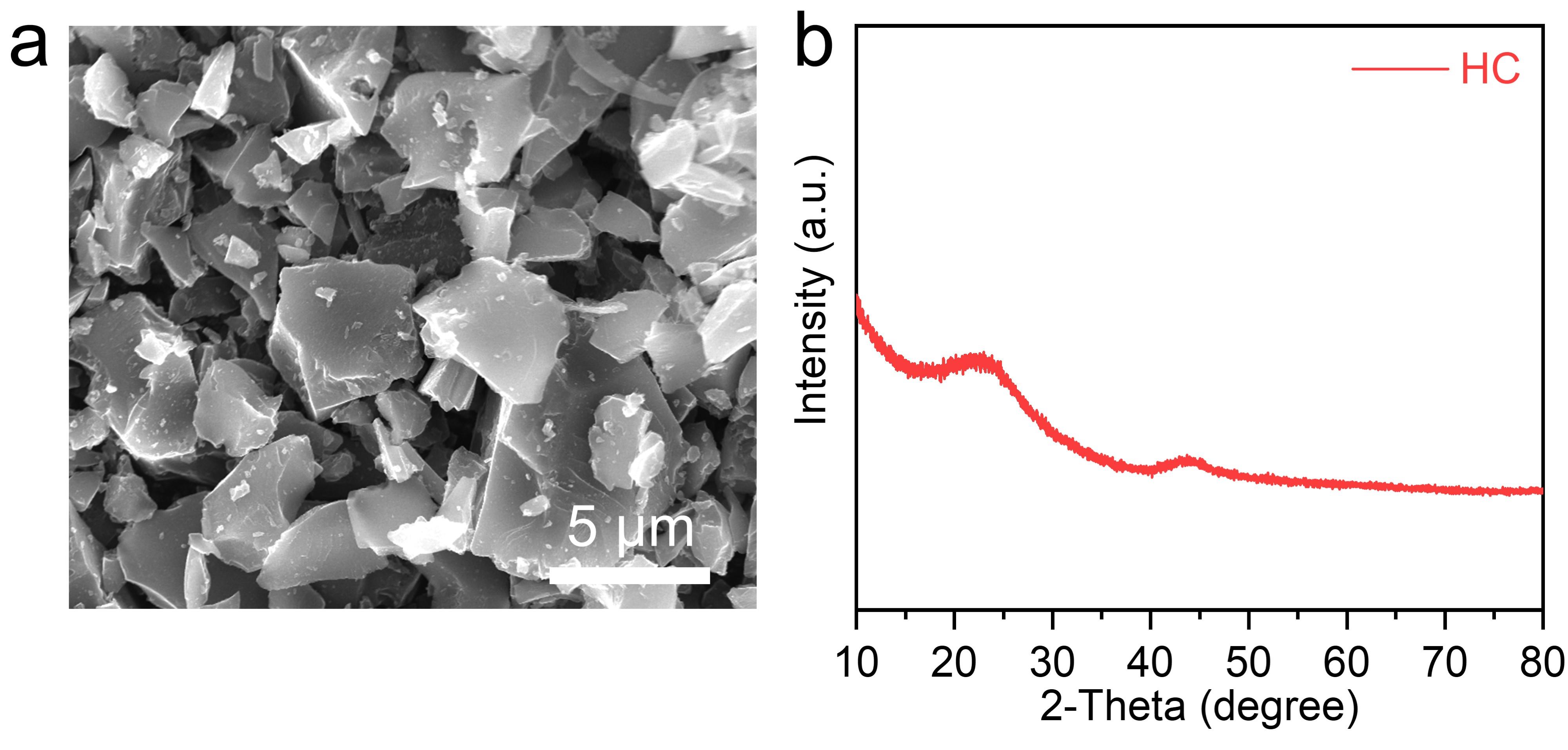


**Figure S30.** The (a) morphology and (b) XRD spectrum of the HC material.


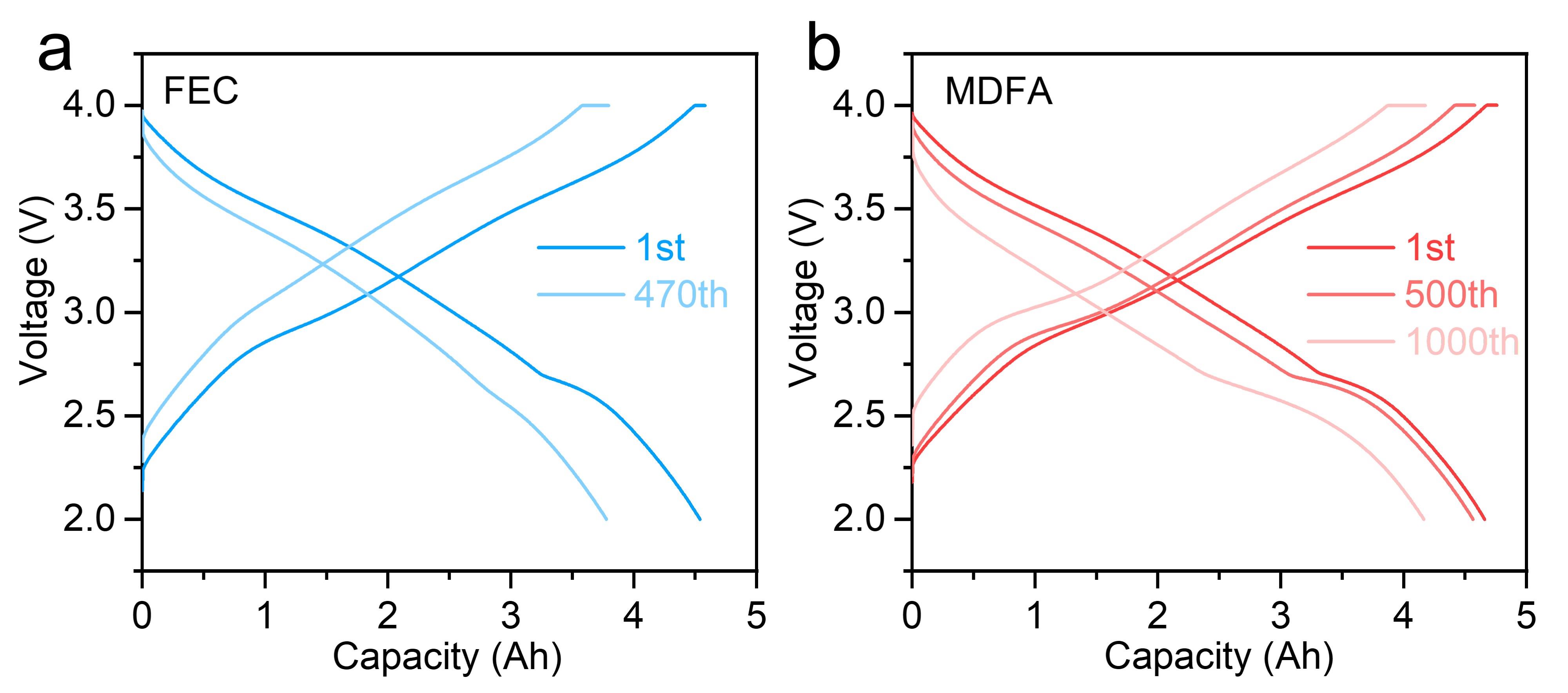


**Figure S31.** The discharging/charging curves in the (a) FEC and (b) MDFA pouch cells.


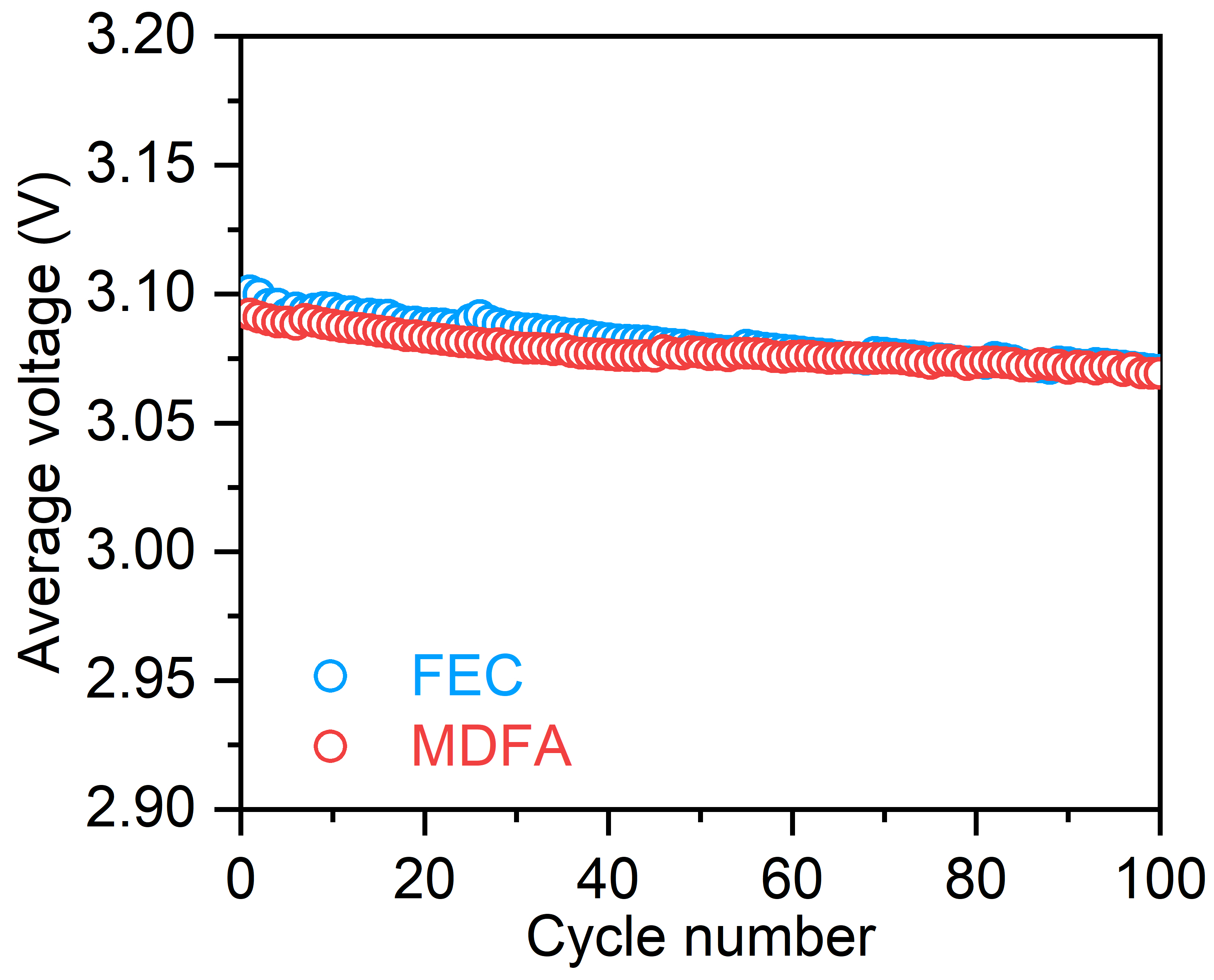


**Figure S32.** The average voltages of pouch cells during 100 cycles.


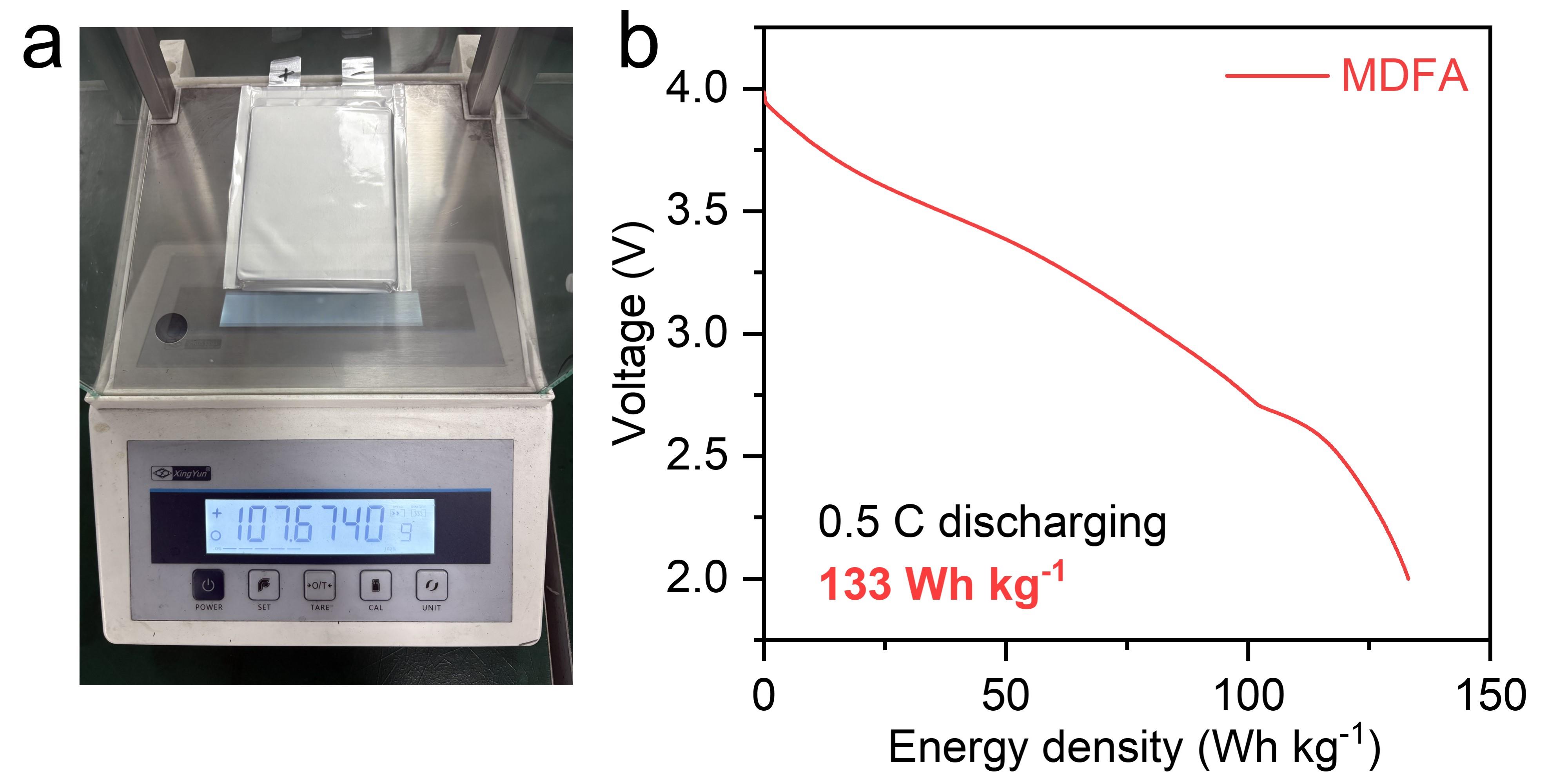


**Figure S33.** The (a) weight and (b) energy density of the MDFA pouch cell.


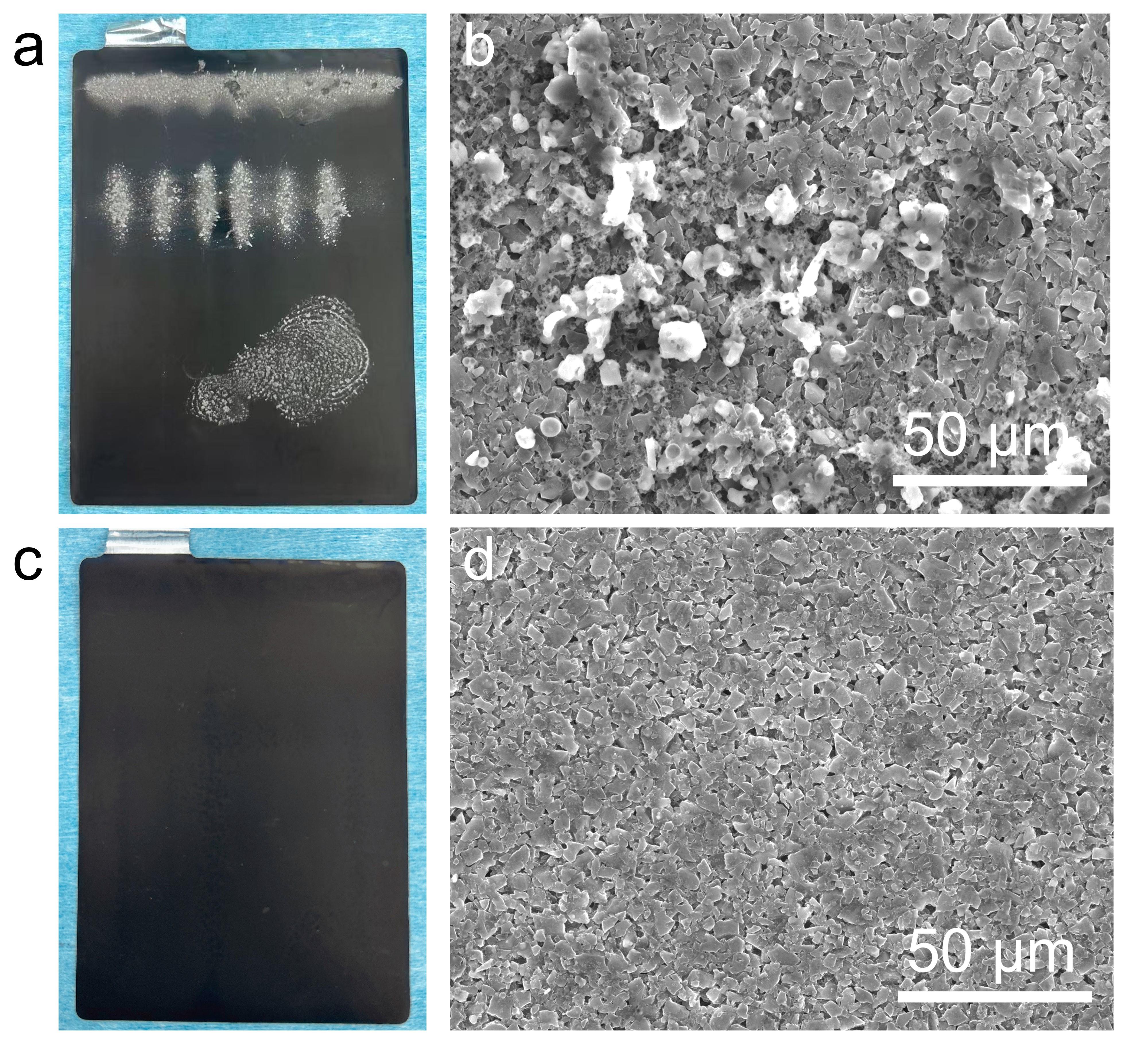


**Figure S34.** The digital photos and SEM images of cycled HC electrodes in the (a, b) FEC and (c, d) MDFA pouch cells.


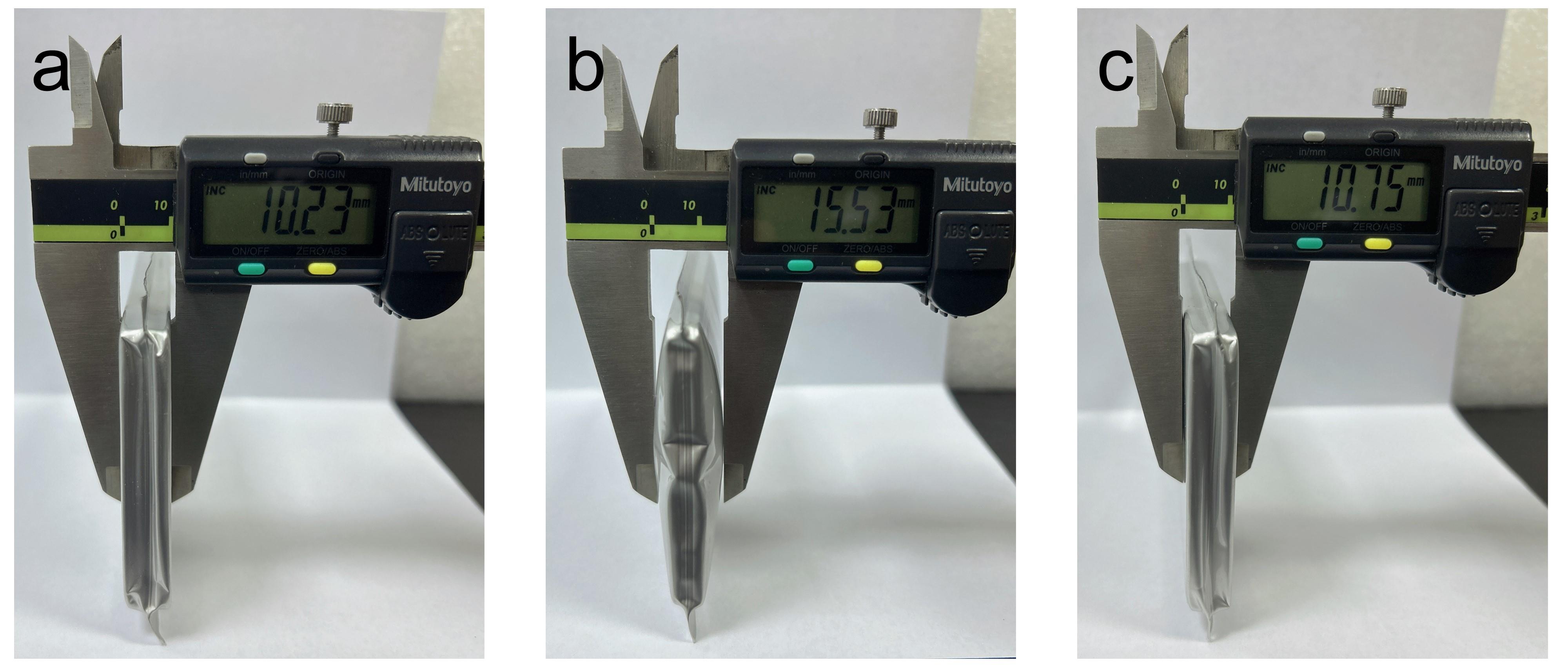


**Figure S35.** The thickness of pouch cells is (a) before cycling, (b) after cycling, in (c) FEC, and (d) MDFA electrolytes.


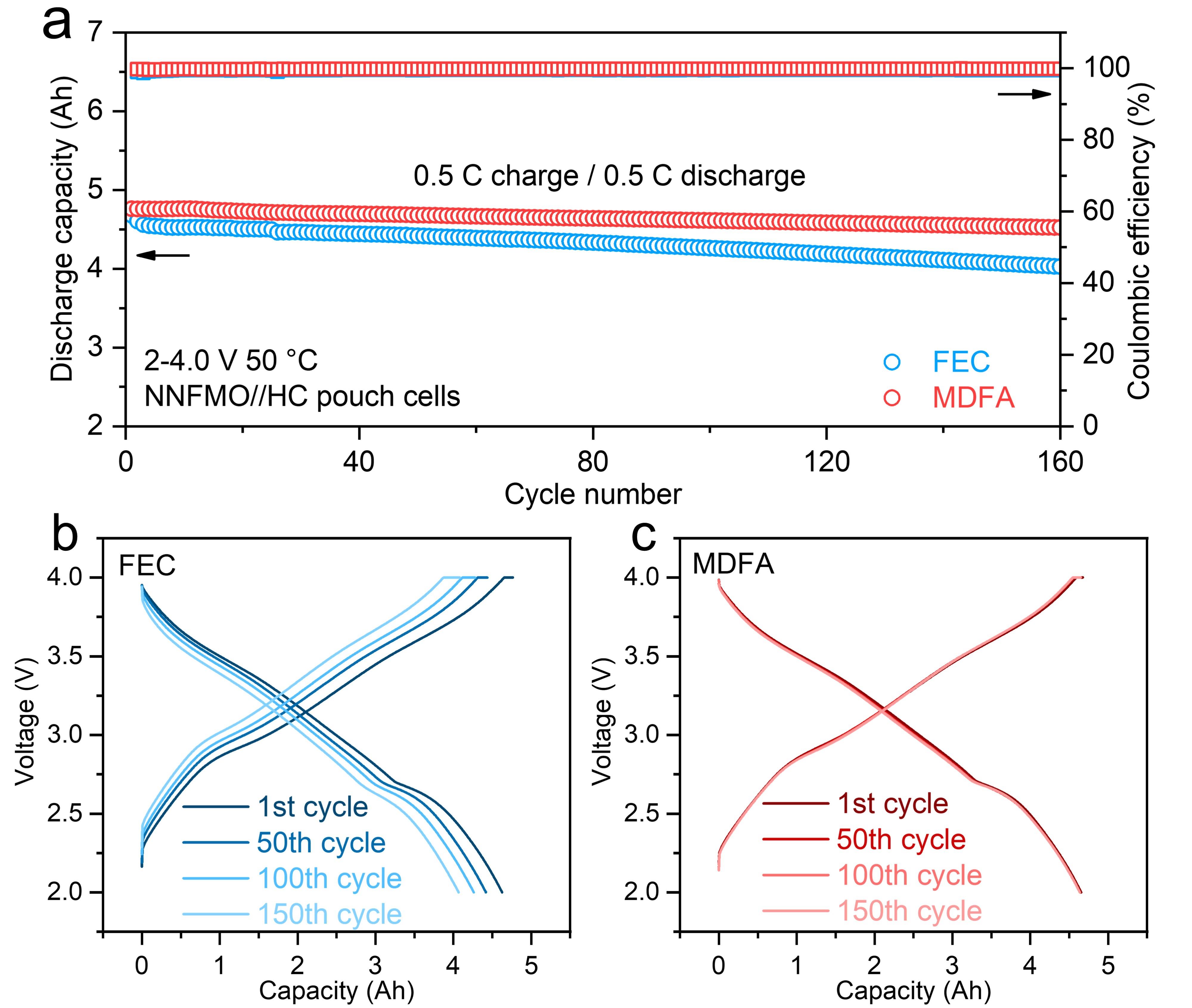


**Figure S36.** (a) The cycling performance of pouch cells at 50 °C. (b) The corresponding discharging/charging curves of the (b) FEC and (c) MDFA cells.


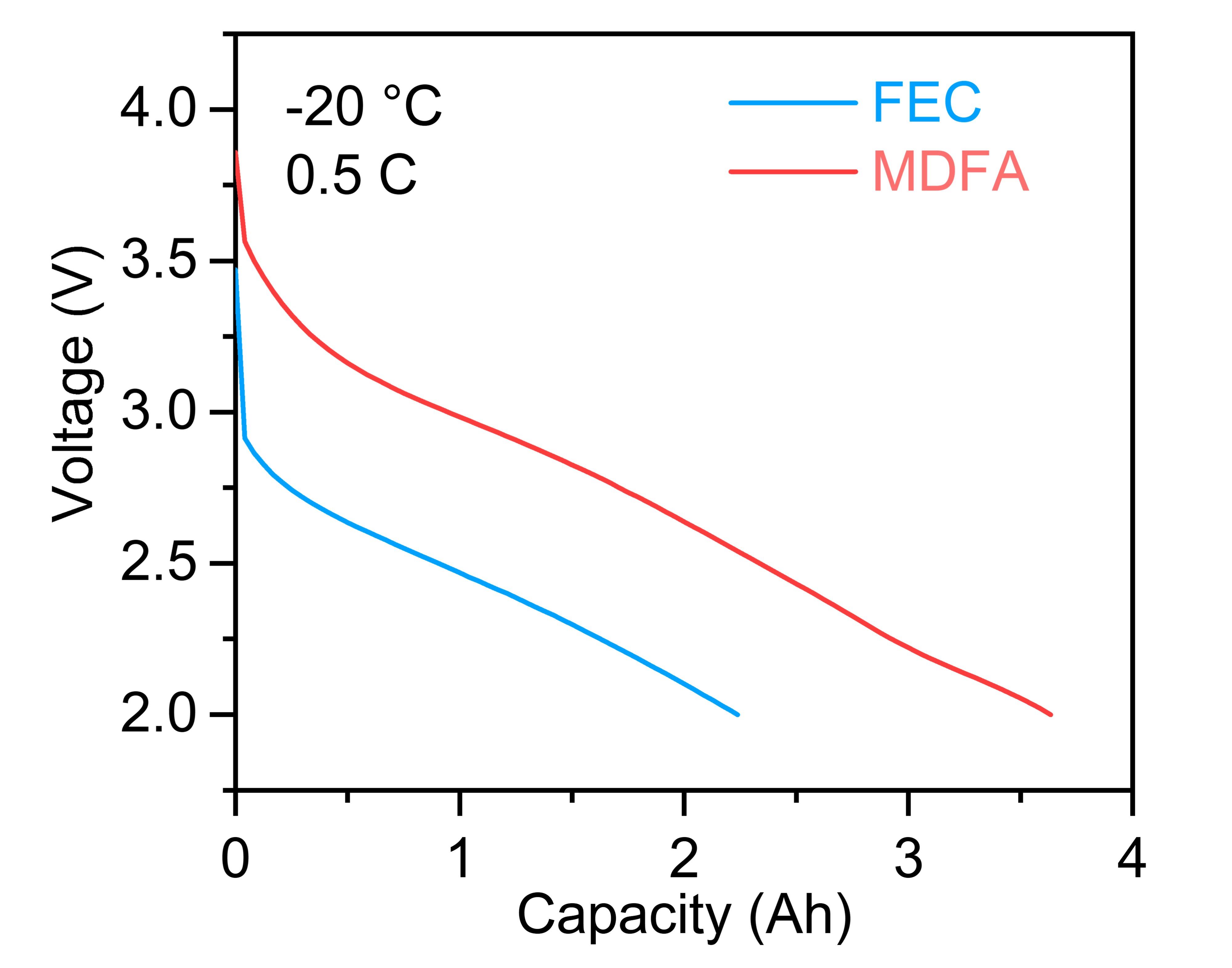


**Figure S37.** The discharge capacity curves of pouch cells at −20 °C.


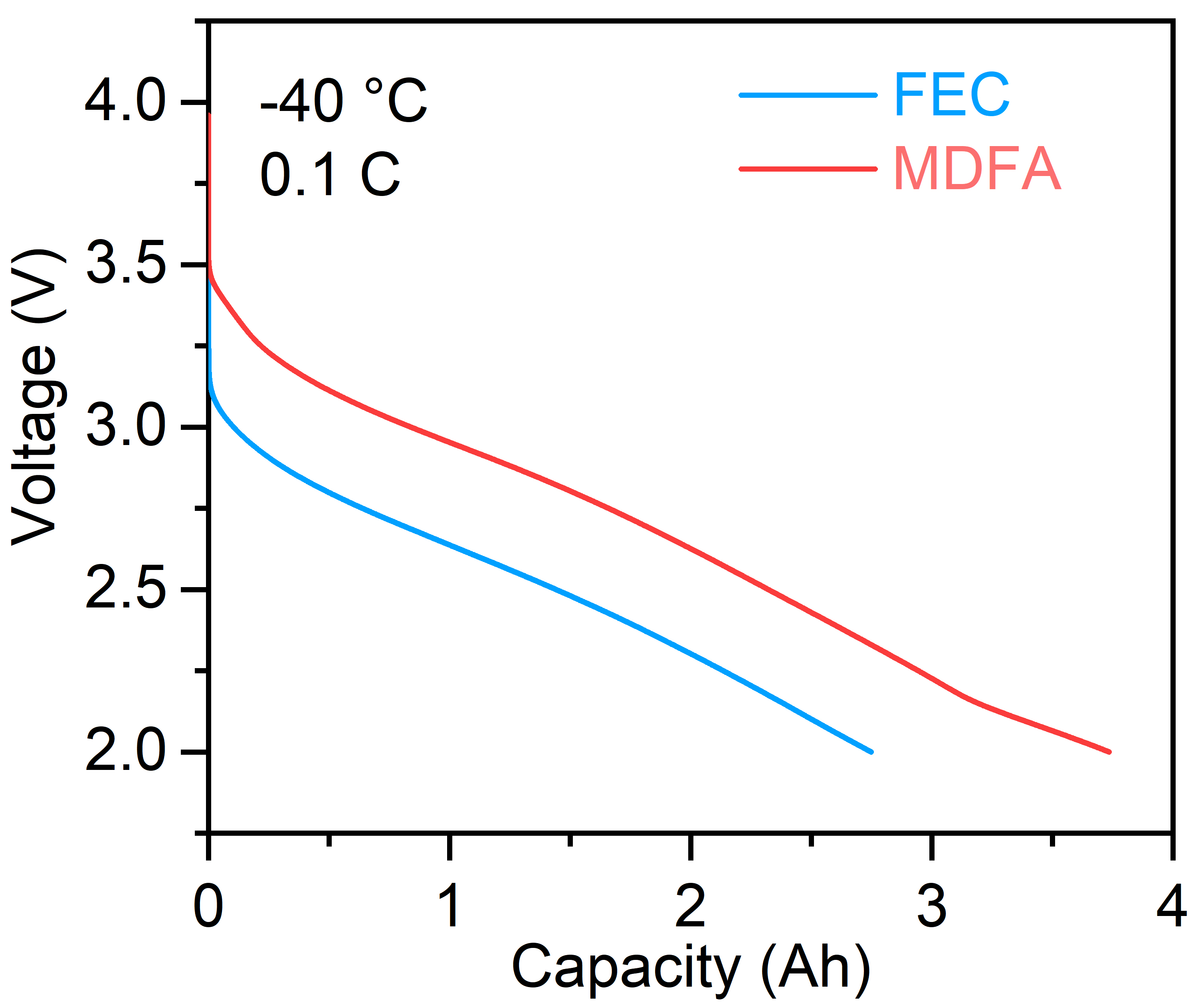


**Figure S38.** The discharging curves of pouch cells at −40 °C and 0.1 C.


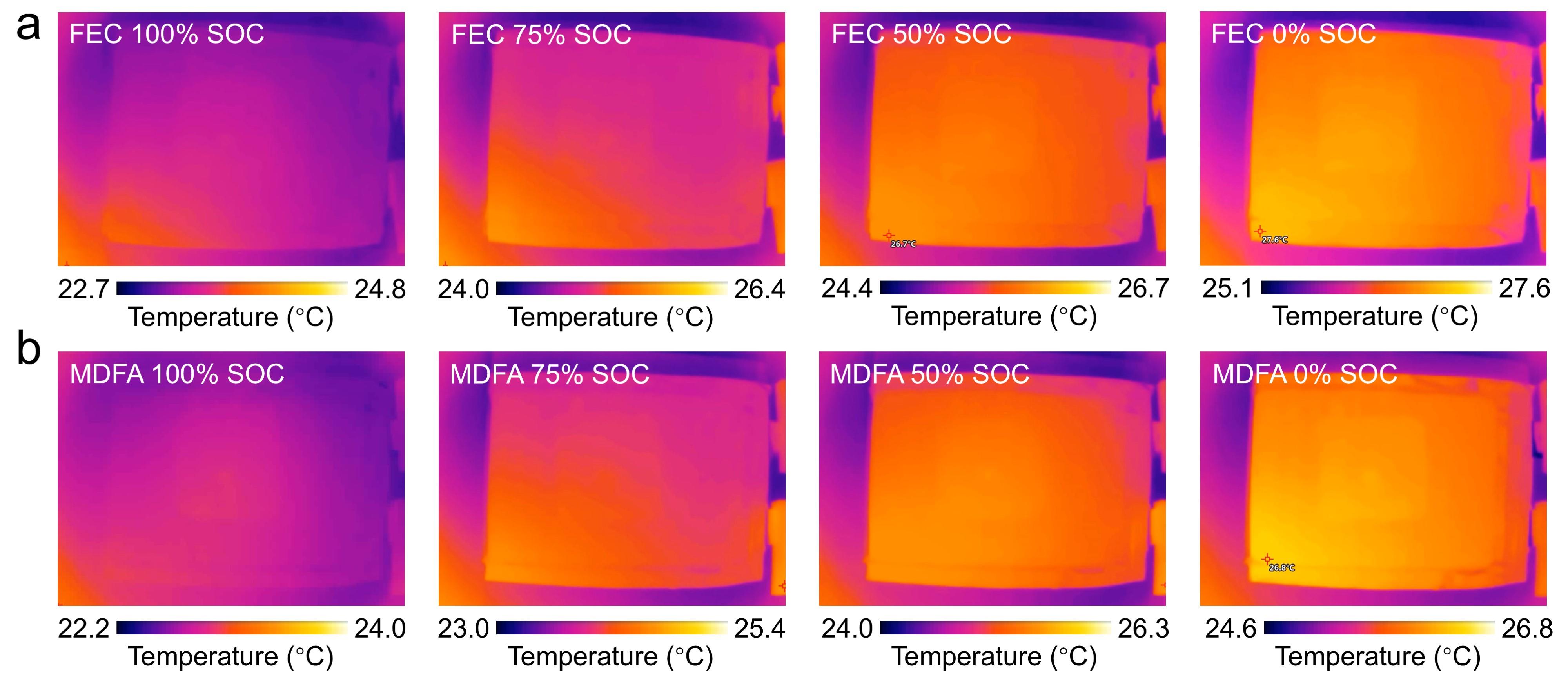


**Figure S39.** The surface temperature of (a) FEC and (b) MDFA pouch cells during 1 C discharging.

**Table S1.** The fabrication parameters of Ah-level NNFMO/HC pouch cells.

| Names | Parameters |  |
| --- | --- | --- |
| Capacity (Ah) | 5 |  |
| Weight (g) | 110 |  |
| Active materials for cathode | NNFMO |  |
| Active materials content in cathode (%) | 95.3 |  |
| Areal mass loading in cathode (mg cm^−2^) | 20.0 |  |
| Areal capacity of cathode (mAh cm^−2^) | 1.93 |  |
| Sizes of cathode (cm) | 60×80 |  |
| Layers of cathode | 25 |  |
| Active materials for anode | HC |  |
| Active materials content in anode (%) | 94.5 |  |
| Areal mass loading in anode (mg cm^−2^) | 8 |  |
| Areal capacity of anode  (mAh cm^−2^) | 2.12 |  |
| Sizes of anode (cm) | | 64×84 |
| Layers of anode | | 26 |
| Thickness of separator (µm) | 13 |  |
| Electrolyte amount (g Ah^−1^) | 4 |  |
